# A promiscuous archaeal cardiolipin synthase enables construction of diverse natural and unnatural phospholipids

**DOI:** 10.1016/j.jbc.2021.100691

**Published:** 2021-04-22

**Authors:** Marten Exterkate, Niels A.W. de Kok, Ruben L.H. Andringa, Niels H.J. Wolbert, Adriaan J. Minnaard, Arnold J.M. Driessen

**Affiliations:** 1 Department of Molecular Microbiology, Groningen Biomolecular Sciences and Biotechnology Institute and Zernike Institute for Advanced Materials, University of Groningen, Groningen, The Netherlands; 2 Department of Chemical Biology, Stratingh Institute for Chemistry, University of Groningen, Groningen, The Netherlands

**Keywords:** cardiolipin, archaea, lipid synthesis, glycolipid, phospholipid, mass spectrometry (MS), membrane protein, glycocardiolipin, cardiolipin synthase, 1Gal-MPCL, monogalactosyl-mono-phosphatidyl-cardiolipin, 1,2-EtOH-DPCL, 1,2-ethanol-di-phosphatidyl-cardiolipin, 1,2-PrOH-DPCL, 1,2-propanediol-di-phosphatidyl-cardiolipin, 1,3-BuOH-DPCL, 1,3-butanediol-di-phosphatidyl-cardiolipin, 1,3-PrOH-DPCL, 1,3-propanediol-di-phosphatidyl-cardiolipin, 1,4-BuOH-DPCL, 1,4-butanediol-di-phosphatidyl-cardiolipin, AA, archaetidic acid, AG, archaetidylglycerol, CDP-DAG, cytidine diphosphate diacylglycerol, CL, cardiolipin (lipid class), Cls, cardiolipin synthase, ClsA, cardiolipin synthase A, ClsB, cardiolipin synthase B, ClsC, cardiolipin synthase C, DDM, n-dodecyl-β-d-maltoside, DOPA, di-oleoyl phosphatidic acid, DOPC, di-oleoyl phosphatidylcholine, DOPE, di-oleoyl phosphatidylethanolamine, DOPG, di-oleoyl phosphatidylglycerol, DOPI, di-oleoyl phosphatidylinositol, DOPS, di-oleoyl phosphatidylserine, glyco-MACL, glycosyl-mono-archaetidyl-cardiolipin, glyco-MPCL, glycosyl-mono-phosphatidyl-cardiolipin, Gro-APCL, glycerol-archaetidyl-phosphatidyl-cardiolipin, Gro-DACL, glycerol-di-archaetidyl-cardiolipin, Gro-DPCL, glycerol-di-phosphatidyl-cardiolipin, LUCA, last universal common ancestor, mannitol-DPCL, mannitol-di-phosphatidyl-cardiolipin, MGDG, monogalactosyldiacylglycerol, MhCls, *Methanospirillum hungatei* cardiolipin synthase, Ni-NTA, Nickel-nitrilotriacetic Acid, P-1-PrOH, phosphatidyl-1-propanol, P-1,2-EtOH, phosphatidyl-1,2-ethanol, P-1,2-PrOH, phosphatidyl-1,2-propandiol, P-1,3-BuOH, phosphatidyl-1,3-butanediol, P-1,3-PrOH, phosphatidyl-1,3-propandiol, P-1,4-BuOH, phosphatidyl-1,4-butanediol, P-2-Phe-1,3-PrOH, phosphatidyl-2-phenyl-1,3-propanediol, P-2-PrOH, phosphatidyl-2-propanol, P-2,2-Me-1,3-PrOH, phosphatidyl-2,2-dimethyl-1,3-propanediol, P-mannitol, phosphatidyl-mannitol, P-NH2-PrOH, phosphatidyl-aminopropanol, PA, phosphatidic acid, PG, phosphatidylglycerol, POPA, palmitoyl-oleoyl phosphatidic acid, POPG, palmitoyl-oleoyl phosphatidylglycerol, S-DGD-5-PA, S-diphytanylglycerol diether-5-phosphatidic acid, S-2Glyco-aMPCL, S-di-glycosyl-archaeal mono-phosphate cardiolipin, S-2Glyco-DGD, S-di-glycosyl diphytanylglycerol diether, S-3Glyco-aMPCL, S-tri-glycosyl-archaeal mono-phosphate cardiolipin, S-GL-2, S-glycosylcardiolipin-2, S-TGD-1-PA, S-tri-glycosyl-diether-1-phosphatidic acid

## Abstract

Cardiolipins (CL) are a class of lipids involved in the structural organization of membranes, enzyme functioning, and osmoregulation. Biosynthesis of CLs has been studied in eukaryotes and bacteria, but has been barely explored in archaea. Unlike the common fatty acyl chain–based ester phospholipids, archaeal membranes are made up of the structurally different isoprenoid-based ether phospholipids, possibly involving a different cardiolipin biosynthesis mechanism. Here, we identified a phospholipase D motif–containing cardiolipin synthase (MhCls) from the methanogen *Methanospirillum hungatei*. The enzyme was overexpressed in *Escherichia coli*, purified, and its activity was characterized by LC-MS analysis of substrates/products. MhCls utilizes two archaetidylglycerol (AG) molecules in a transesterification reaction to synthesize glycerol-di-archaetidyl-cardiolipin (Gro-DACL) and glycerol. The enzyme is nonselective to the stereochemistry of the glycerol backbone and the nature of the lipid tail, as it also accepts phosphatidylglycerol (PG) to generate glycerol-di-phosphatidyl-cardiolipin (Gro-DPCL). Remarkably, in the presence of AG and PG, MhCls formed glycerol-archaetidyl-phosphatidyl-cardiolipin (Gro-APCL), an archaeal-bacterial hybrid cardiolipin species that so far has not been observed in nature. Due to the reversibility of the transesterification, in the presence of glycerol, Gro-DPCL can be converted back into two PG molecules. In the presence of other compounds that contain primary hydroxyl groups (*e.g.*, alcohols, water, sugars), various natural and unique unnatural phospholipid species could be synthesized, including multiple di-phosphatidyl-cardiolipin species. Moreover, MhCls can utilize a glycolipid in the presence of phosphatidylglycerol to form a glycosyl-mono-phosphatidyl-cardiolipin species, emphasizing the promiscuity of this cardiolipin synthase that could be of interest for bio-catalytic purposes.

Cardiolipin (CL) is present in lipid membranes throughout all three domains in life. This lipid class comprises lipid species that contain two pairs of lipid tails that are each linked to a glycerol(phosphate) backbone, with a bridging polar headgroup. 1,3-bis(*sn*-3’-phosphatidyl)-*sn*-glycerol or simply glycerol-di-phosphatidyl-cardiolipin (Gro-DPCL) is the most prominent and studied cardiolipin species. Gro-DPCL is usually a relatively minor component (<10 mol%) of the total membrane lipid composition, and its primary role appears to be supporting the function of various membrane proteins (1, 2). Unlike most naturally occurring glycerophospholipids, Gro-DPCL consists of two 1,2-diacyl-glycerolphosphate moieties esterified to the 1- and 3-hydroxyl groups of a glycerol molecule. Because of the two phosphates, it can carry up to two negative charges (3). Furthermore, the polar head group is relatively small compared with the four bulky hydrophobic tails, giving Gro-DPCL its characteristic inverted conical shape in the presence of divalent cations (4). Due to this structural feature, Gro-DPCL may induce membrane curvature. Indeed, Gro-DPCL is believed to be located in lipid domains at the cell poles and division site in bacteria (5, 6, 7, 8), and in eukaryotes, it is an important constituent of the curvy mitochondrial membrane (9, 10, 11). Besides a tight association with cytochrome *c* oxidase, a part of the respiratory chain complex (12, 13, 14), other specific Gro-DPCL–protein interactions seem not to be conserved among the three domains of life. In bacteria, Gro-DPCL is often not an essential membrane constituent, and in several organisms its production is upscaled under certain specific circumstances, such as during stationary phase, or when cells are exposed to certain stressors, *e.g.*, osmotic shock (15, 16, 17, 18).

Cardiolipins have also been described in archaea, most notably in Euryarchaeota. Specifically, in halophiles the production of glycerol-di-archaetidyl-cardiolipin (Gro-DACL) is influenced by changes in the ionic composition of the environment (19). For instance, in the halophilic organism *Halorubrum* sp., hypotonic stress resulted in increased production of Gro-DACL, as well as the mono-archaetidyl-cardiolipin species S-di-glycosyl-mono-archaetidyl-cardiolipin (S-2glyco-MACL), which only contains one phosphate group. This cardiolipin species (originally referred to as: S-DGD-5-PA) consists of an archaetidic acid (AA) molecule attached to a sulfated diglycosyl diphytanylglycerol diether (S-2Glyco-DGD) (20). Further, a variety of other glycosyl-mono-archaetidyl-cardiolipin (glyco-MACL) species have been identified in archaea (19). For example, *Halobacterium salinarum* produces an S-tri-glycosyl-diether glycolipid fused to AA (S-3Glyco-MACL, originally referred to as: S-TGD-1-PA) (21), while *Haloferax volcanii* also contains the glycosyl cardiolipin analogue S-2Glyco-MACL (originally referred to as: S-GL-2) (22). The polar head group of these glyco-MACL species is structurally very different from the classical di-phosphatidyl/archaetidyl cardiolipin species, which raises questions on the specific function of these lipids, as well as the enzymatic mechanism of their synthesis.

Whereas cardiolipin synthesis in Eukaryotes and Bacteria has been studied extensively, the mechanism of cardiolipin biosynthesis in Archaea has largely remained unexplored. Currently, two phylogenetically distinct types of enzyme families are known in Bacteria and Eukaryotes to synthesize di-phosphatidyl-cardiolipin. One family is characterized by the presence of a cytidine diphosphate (CDP)-alcohol phosphatidyltransferase domain (Cls_cap), which catalyzes Gro-DPCL synthesis by utilizing the substrates cytidine diphosphate diacylglycerol (CDP-DAG) and phosphatidylglycerol (PG) (23). In this reaction, the first phosphate group connected to DAG is coupled to the polar glycerol head of PG, and a cytidine monophosphate (CMP) is released. Cls_cap enzymes are predominantly found in eukaryotes, but have more recently also been identified in bacteria (24). The second family features two phospholipase D (PLD) domains (Cls_pld), which enable Gro-DPCL synthesis by transferring the phosphatidyl group, originating from a PG molecule, to another PG, whereby a glycerol molecule is released. This type of Gro-DPCL biosynthesis is predominantly present in Bacteria, but has been found in eukaryotes as well (25, 26). This latter reaction is catalyzed by the ClsA and ClsB-type cardiolipin synthases (Cls) (27, 28). However, an alternative synthesis method has been identified in *Escherichia coli* in which phosphatidylethanolamine (PE) is used together with PG, to form Gro-DPCL and ethanolamine, a reaction catalyzed by the PLD-containing ClsC-type enzymes (17, 29). Homology searches in genomes of archaea revealed Cls-like members from both Cls_pld and Cls_cap cardiolipin synthesizing families. However, until now no archaeal enzyme has been experimentally associated with any cardiolipin biosynthesis. Here, we report on the identification and characterization of a PLD-containing cardiolipin synthase of the Euryarchaeote *Methanospirillum hungatei*. The enzyme showed a remarkable promiscuity with respect to accepted and produced lipid species, ranging from a wide variety of phospholipids and di-phosphatidyl/archaetidyl-cardiolipins to include even glycolipid and glycosyl-mono-phosphatidyl-cardiolipin species.

## Results

### Bioinformatic identification of cardiolipin synthases in archaea

**Figure 1:**
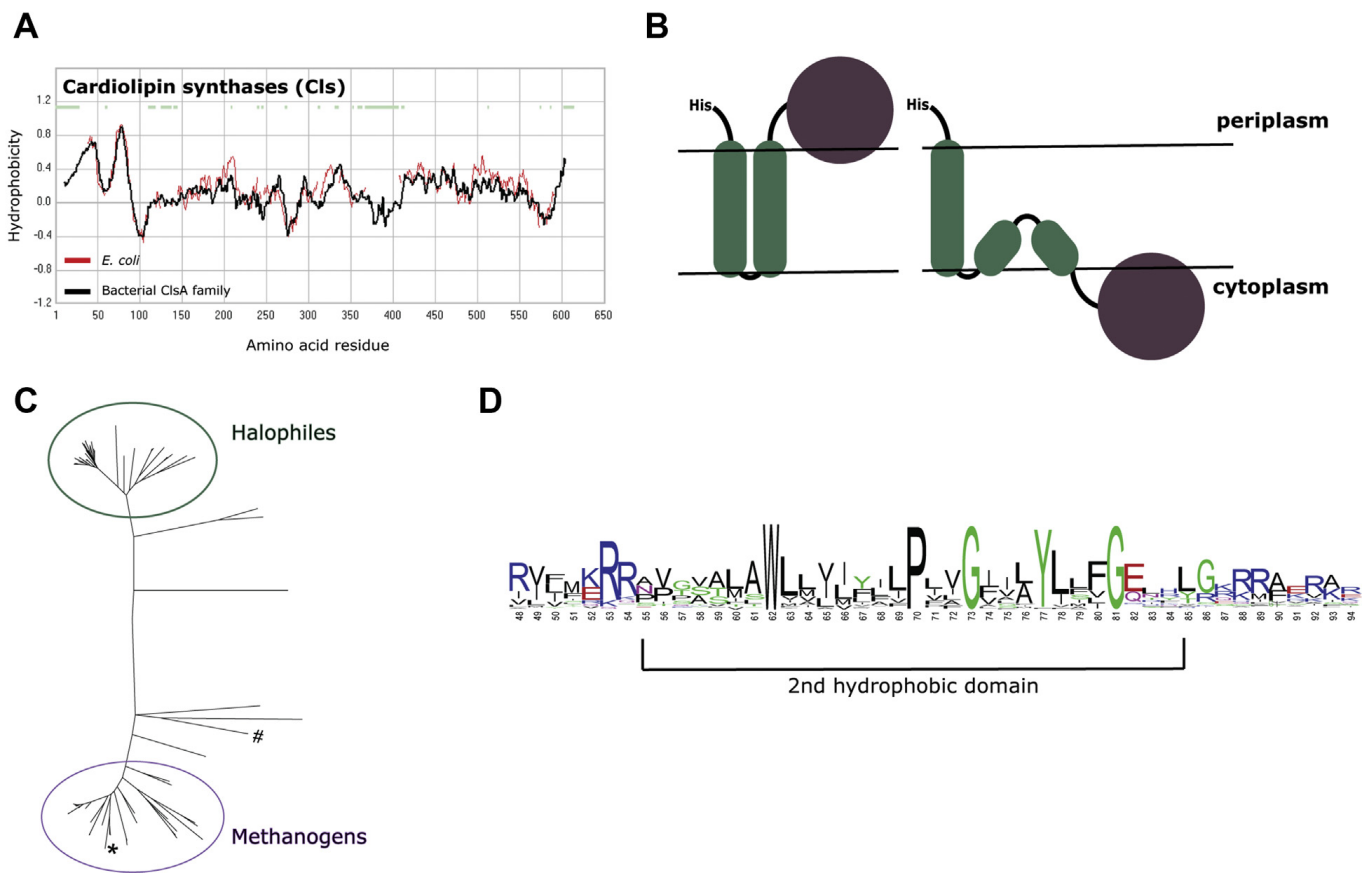
**Bioinformatic identification of an archaeal cardiolipin synthase (Cls).***A*, hydropathy profile alignment of *E. coli* ClsA (*red line*), with the averaged hydropathy profile of its bacterial protein family (*black line*). *B*, schematic representation of potential membrane topologies of cardiolipin synthase with two predicted transmembrane anchors and a globular active domain. *C*, unrooted tree of putative archaeal cardiolipin synthases with two main clusters. # *E. coli* ClsA; ∗ *M. hungatei* Cls. A detailed tree can be found in the Fig. S1. *D*, consensus sequence logo of the second hydrophobic region of cardiolipin synthase type A enzymes from bacteria and the group of methanogenic archaea.

Cardiolipins have only been identified in some archaea, most notably in halophiles: a group of organisms belonging to the phylum of the Euryarchaeota (19). To identify possible cardiolipin synthesizing enzymes in archaea, we performed a BLAST homology search with both Cls_cap and Cls_pld templates to the domain of Archaea. For the identification of archaeal Cls_cap candidates, the cardiolipin synthase from *Streptomyces coelicolor A3* (Sco1389) was used, for which mostly hypothetical proteins were found that could only be appointed to a specific (sub-)phylum (*e.g.*, candidatus Woesearchaeota archaeon) (data not shown) (30). Although these hypothetic proteins contain the cap-motif, no cultured individual archaeal species was identified, and therefore they were not further used in this study. Likewise, to identify putative PLD-containing archaeal cardiolipin synthases, the three Cls enzymes (ClsA, ClsB, and ClsC) of the bacterium *E. coli* were used as template in the BLAST search. This resulted in similar hits, with the best sequence coverage and identity with ClsA (see Supporting information). After comparing the BLAST results obtained for all three Cls_pld templates, the sequence coverage and identity with *E. coli* ClsA (EcClsA) were the largest, so it was decided to continue with these putative archaeal candidates. Although the crystal structure of EcClsA has not been determined, a membrane topology prediction based on the average hydropathy profile of the amino acid sequences of a family of bacterial ClsA proteins reveals the presence of two hydrophobic regions at the N-terminus of this membrane protein (corresponding to amino acids 7–29 and 39–61 in ClsA) (Fig. 1*A*). These could represent transmembrane segments that are linked to a C-terminal globular domain (Fig. 1*B*, left panel) that contains two HKD motifs (HxKx_4_D), which are universally present in cardiolipin synthases that belong to the phospholipase D superfamily (28). The BLAST homology search revealed two main clusters of archaeal homologs that also contain the two HKD motifs (Fig. 1*C*), which are discussed in more detail in the Supporting information (Figs. S1 and S2). One cluster consists of halophilic archaea and the other concerns methanogenic archaea, both groups belonging to the Euryarchaeota. The enzymes from the cluster of methanogenic archaea show about 20 to 25% sequence identity with the *E. coli* ClsA, including the predicted two hydrophobic regions at the N-terminus (Fig. S3). Specifically, the second predicted N-terminal hydrophobic domain contains the conserved motif: Wx_7_Px_2_Gx_3_Yx_3_G (“x” represents a hydrophobic amino acid), present also in bacterial ClsA-type proteins (Fig. 1*D*), but not found in enzymes belonging to the halophilic cluster (Fig. S2). The high incidence of hydrophobic amino acids suggests that this region is likely embedded in the membrane. The conserved proline and glycine (PxxG) residues are located in the middle of the hydrophobic stretch. These two amino acids are often found in turns and loops and thus may introduce flexibility in this predicted helix region (31, 32). Furthermore, this hydrophobic region is flanked on both sides by multiple positive charges, which are known to inhibit translocation across the membrane according to the positive-inside rule and thus may affect the membrane topology of this region. As a consequence, this predicted helix domain may not span across the membrane, but represent a re-entrance loop that enters and leaves the membrane at the same leaflet side (Fig. 1*B*, right panel). Taken together, this group of methanogenic ClsA-like proteins all appear to be promising candidates for potential archaeal cardiolipin synthases. The putative *M. hungatei* Cls (WP_011448254) was selected for further characterization as it is the only candidate present in this organism. Furthermore, it grows at mesophilic temperatures and a pH around 7, with an osmotic requirement that is comparable to *E. coli*, which was used for protein overexpression.

### Glycerol-di-archaetidyl-cardiolipin (Gro-DACL) synthesis from archaetidylglycerol (AG)

**Figure 2:**
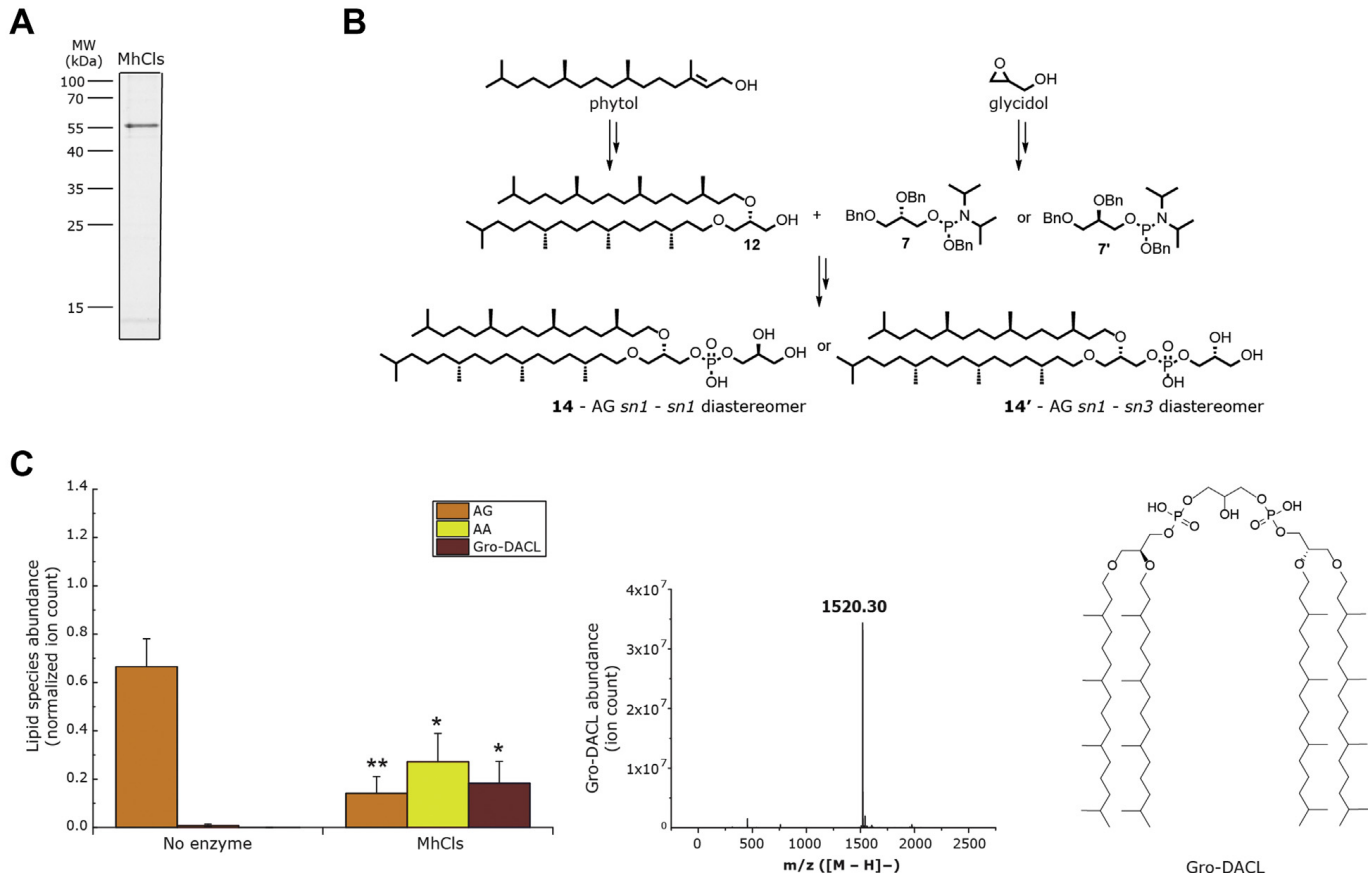
**Purification and activity of the cardiolipin synthase from *M. hungatei* (MhCls).***A*, Coomassie stained SDS-PAGE gel of the MhCls purified by Ni-NTA chromatography. *B*, schematic representation of chemical archaetidylglycerol (AG) *sn*1-*sn*1 **(14)**, and *sn*1-*sn*3 **(14’)** synthesis. *C*, *in vitro* activity of purified MhCls. AG was incubated overnight (18 h) in the absence and presence of the enzyme MhCls. Lipid species were analyzed by LC-MS. Levels of AG, archaetidic acid (AA), and glycerol-di-archaetidyl-cardiolipin (Gro-DACL) were normalized for the internal standard DDM and plotted on the y-axis. Data are mean ± SD (n = 3). Statistical significance is shown for the enzymatic reaction (MhCls) compared with the control (no enzyme), for each individual lipid species, by using the Student’s *t*-test analysis; ∗*p* ≤ 0.05; ∗∗*p* ≤ 0.01. Mass spectrum showing the presence of Gro-DACL as (*m/z* 1520.30 [M-H]^−^) and its structure.

The putative *M. hungatei* ClsA homologue (MhCls) was ordered as an *E. coli* codon-optimized synthetic gene, cloned into a His-tag containing overexpression vector, and expressed in *E. coli*. Overexpressed MhCls was recovered from the membrane fraction, solubilized with the detergent n-dodecyl-β-d-maltoside (DDM; 2%, w/v), and purified by Ni-NTA agarose affinity chromatography (Fig. 2*A*). To confirm that MhCls is indeed an archaeal cardiolipin synthase, the activity of purified enzyme toward the substrate archaetidylglycerol (AG) was tested *in vitro*. Since this archaeal equivalent of PG is not commercially available, both the *sn*1-*sn*1 **(14)** and the *sn*1-*sn*3 **(14’)** diastereomers of AG were chemically synthesized (Fig. 2*B*) (see the Supporting information; Figs. S4–S6). The bis-phytanyl glycerol core was readily synthesized from commercially available phytol and glycidol. Both enantiomers of the glycerol-phosphate headgroup were also produced by asymmetric synthesis and were individually coupled to the aforementioned chiral lipid core *via* a phosphor–amidite coupling. After the successful synthesis of *sn1-sn1* and *sn1-sn3* AG, first a mixture of both diastereomers was incubated overnight at 37 °C together with purified MhCls. LC-MS analysis revealed that the majority of AG was consumed, which coincided with the production of an ion *m/z* 1520.30 [M-H]^−^, corresponding to glycerol-di-archaetidyl-cardiolipin (Gro-DACL) (Fig. 2*C*; for fragmentation data see Fig. S7). In the absence of MhCls, no such conversion was observed. Subsequently the activity of MhCls toward the individual AG diastereomers (*sn1* and *sn3*) was tested and compared, but both acted as substrates in a similar manner (data not shown). Aside from producing Gro-DACL, MhCls additionally synthesized the lipid species archaetidic acid (AA) (Fig. 2*C*). The latter is probably the result of an unsuccessful transfer of the archaetidyl group, whereby the enzyme hydrolyzes the terminal phosphodiester bond in a phospholipase D-like manner.

**Figure 3:**
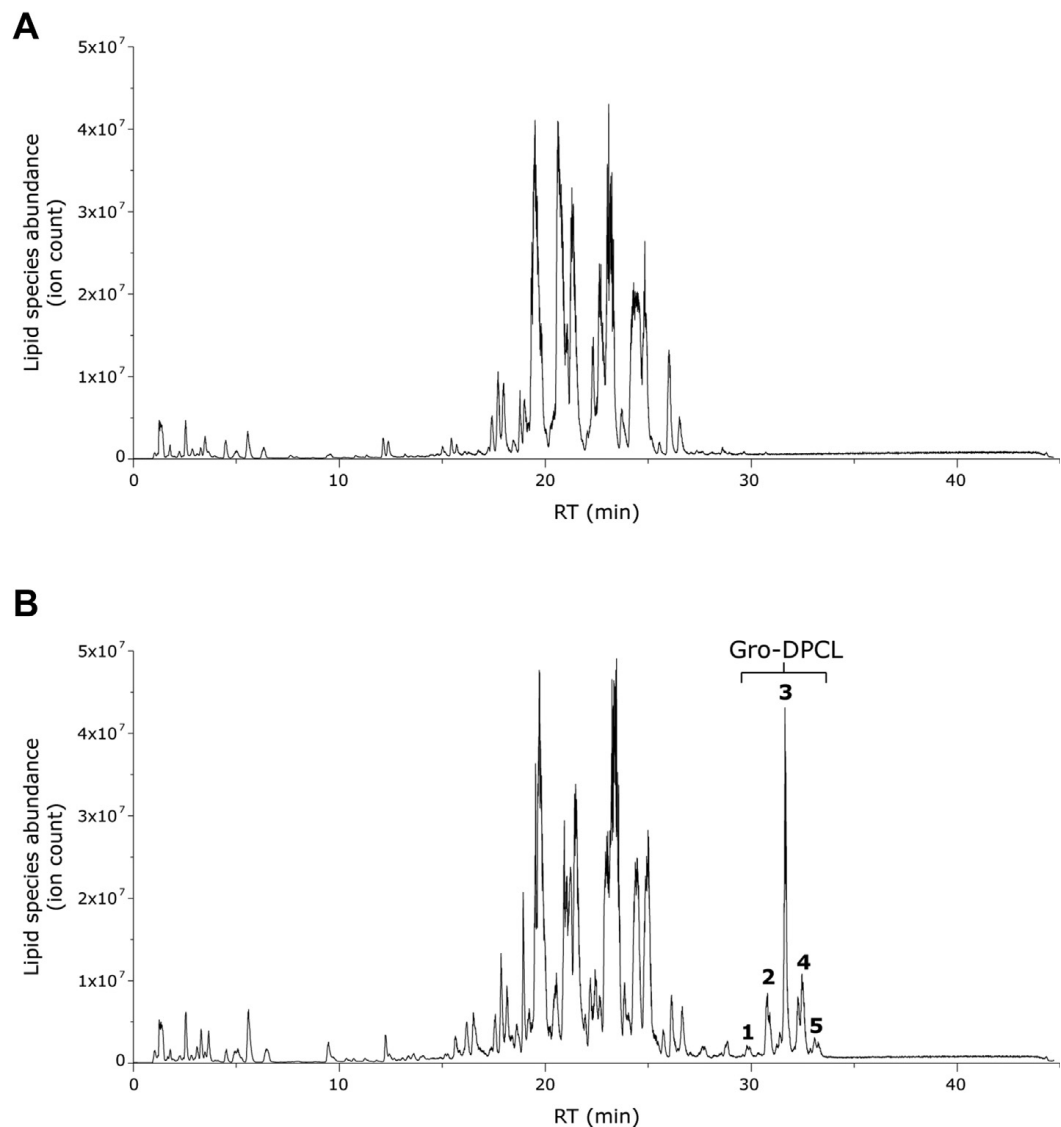
**Gro-DPCL biosynthesis by MhCls expressed in the *E. coli clsABC null* strain.** LC-MS chromatograms of (*A*) lipidome of the *E. coli clsABC null* strain overexpressing an empty vector, showing a wide variety of lyso-phospholipids (RT 0–8 min) and di-acyl phospholipids (RT 17–23 min) (*B*) Lipidome of the *E. coli clsABC null* strain overexpressing MhCls, showing the additionally formed glycerol-di-phosphatidyl-cardiolipin (Gro-DPCL) species (RT 29–34 min), that elute in five main peaks. Predominantly cardiolipin species present in peak 1: Gro-DPCL 60:2 (*m/z* 1291.87 [M-H]^−^); peak 2: Gro-DPCL 62:2 (*m/z* 1319.90 [M-H]^−^); peak 3: Gro-DPCL 64:2 (*m/z* 1347.93 [M-H]^−^); peak 4: Gro-DPCL 66:2 (*m/z* 1375.96 [M-H]^−^); peak 5: Gro-DPCL 67:2 (*m/z* 1389.98 [M-H]^−^).

To verify that MhCls functions as a cardiolipin synthase *in vivo*, the gene was expressed in an *E. coli clsABC* null strain (17). Subsequent analysis of the lipidome revealed the presence of multiple Gro-di-phosphatidyl-cardiolipin (Gro-DPCL) species (differing in acyl-chain composition), indicating that this enzyme can also utilize phosphatidyl-containing lipids (Fig. 3*B*). As overexpression of an empty vector resulted in the complete absence of any CL-species (Fig. 3*A*), it can be concluded that MhCls is a cardiolipin synthase.

### A glycerol-dependent dynamic equilibrium between PG and Gro-DPCL

**Figure 4:**
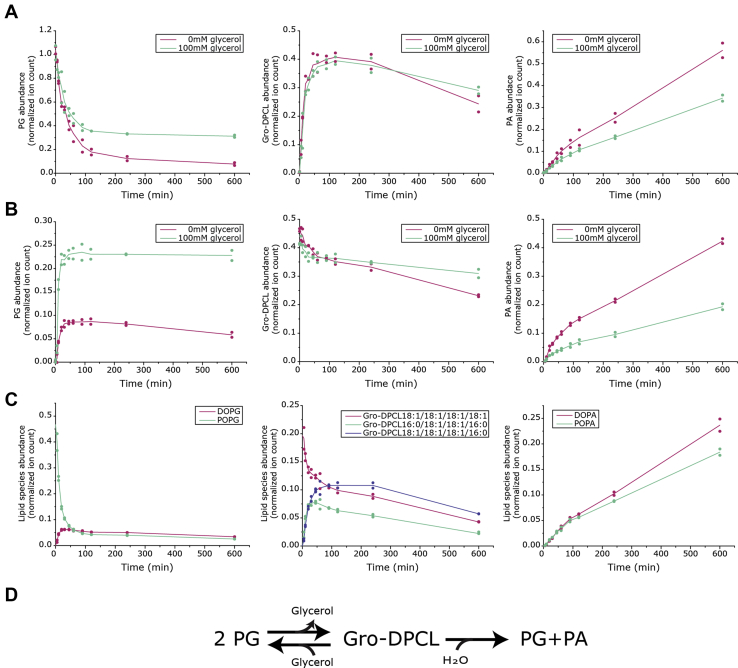
**MhCls activity in the presence or absence of glycerol starting with the substrate(s).***A*, Di-oleoyl-phosphatidylglycerol (DOPG), (*B*) glycerol-di-phosphatidyl-cardiolipin (Gro-DPCL) 18:1/18:1/18:1/18:1, and (*C*) palmitoyl-oleoyl-phosphatidylglycerol (POPG) together with Gro-DPCL 18:1/18:1/18:1/18:1. The formed lipid species PG, Gro-DPCL, and phosphatidic acid (PA) were analyzed by LC-MS, normalized for the internal standard DDM, and ion counts are plotted on the y-axis. *Lines* represent the mean of the data points (n = 2). *D*, schematic representation of the MhCls-mediated glycerol-dependent dynamic equilibrium in Gro-DPCL formation.

Next, the activity of MhCls toward the bacterial equivalents of the archaeal lipids was further tested *in vitro*. To perform the measurements under optimal conditions, adequate membrane reconstitution of MhCls is required for which a bacterial lipid/detergent molar ratio profile was recorded for the substrate PG (Fig. S8). Subsequently, MhCls activity was monitored in the presence of PG, which resulted in the formation of Gro-DPCL, showing again that the enzyme accepts both bacterial and archaeal phospholipids as a substrate. Similar to AG, utilization of PG resulted in the production of not only Gro-DPCL, but also phosphatidic acid (PA). Initially most of the PG is converted into Gro-DPCL, although some PA is produced as well (Fig. 4*A*, purple lines). Eventually the PG levels reach a plateau, and Gro-DPCL levels start to drop, concomitantly with the continuous production of PA. In the presence of a high concentration (100 mM) of glycerol (Fig. 4*A*, green lines), the initial conversion of PG into Gro-DPCL is similar, but production of PA is significantly reduced, while PG reaches a plateau level at a higher concentration. This indicates that glycerol most likely stimulates the formation of PG in a reverse transesterification reaction and thereby competes with the hydrolysis of Gro-DPCL (Fig. 4*D*). To confirm this hypothesis, the same reaction was performed with Gro-DPCL as substrate. In the presence of 100 mM glycerol (Fig. 4*B*, green lines), Gro-DPCL is initially predominantly converted into PG, while only low levels of PA are noted. In contrast, in the absence of glycerol, PA formation is stimulated, while lower levels of PG are observed (Fig. 4*B*, purple lines). These data demonstrate that PA is formed together with PG by hydrolysis of Gro-DPCL (Fig. 4*D*).

Hydrolysis of a Gro-DPCL should yield stoichiometric amounts of PA and PG, but released PG can be directly reutilized for production of this cardiolipin species. To examine the reaction in more detail and to further address the transesterification step, PG and Gro-DPCL, both with a different acyl-chain composition, were introduced. This enables the detection of a single lipid type as a substrate or a product, based on the detected mass. Incubation of MhCls with an equimolar ratio of palmitoyl-oleoyl phosphatidylglycerol (POPG 16:0/18:1) and di-oleoyl-di-oleoyl-glycerol-di-phosphatidyl-cardiolipin (Gro-DPCL 18:1/18:1/18:1/18:1) resulted in the expected production of palmitoyl-oleoyl-palmitoyl-oleoyl-glycerol-di-phosphatidyl-cardiolipin (Gro-DPCL 16:0/18:1/18:1/16:0), di-oleoyl phosphatidylglycerol (DOPG 18:1/18:1), and di-oleoyl phosphatidic acid (DOPA 18:1/18:1), but also in the production of palmitoyl-oleoyl phosphatidic acid (POPA 16:0/18:1), demonstrating that the enzymatic reaction occurs in both directions (Fig. 4*C*). Moreover, a Gro-DPCL species with the mixed 16:0/18:1 and 18:1/18:1 acyl-chain configuration could be identified as well, which is the synthesis product of POPG combined with DOPG. The latter originated from the reverse-transesterification reaction of Gro-DPCL 18:1/18:1/18:1/18:1, thereby showing the dynamic character of the PG - Gro-DPCL equilibrium (Fig. 4*D*). On the other hand, the accumulation of PA suggests that this reaction eventually becomes unidirectional as PA cannot be further used. Indeed, when POPA 16:0/18:1 was added to a reaction containing DOPG 18:1/18:1, the MhCls-mediated reaction resulted in the formation of only Gro-DPCL 18:1/18:1/18:1/18:1 and DOPA, whereas no POPG or Gro-DPCL 16:0/18:1/16:0/18:1 was detected, showing that PA cannot be reutilized by MhCls (Fig. S9).

### Formation of a bacterial–archaeal hybrid cardiolipin species

**Figure 5:**
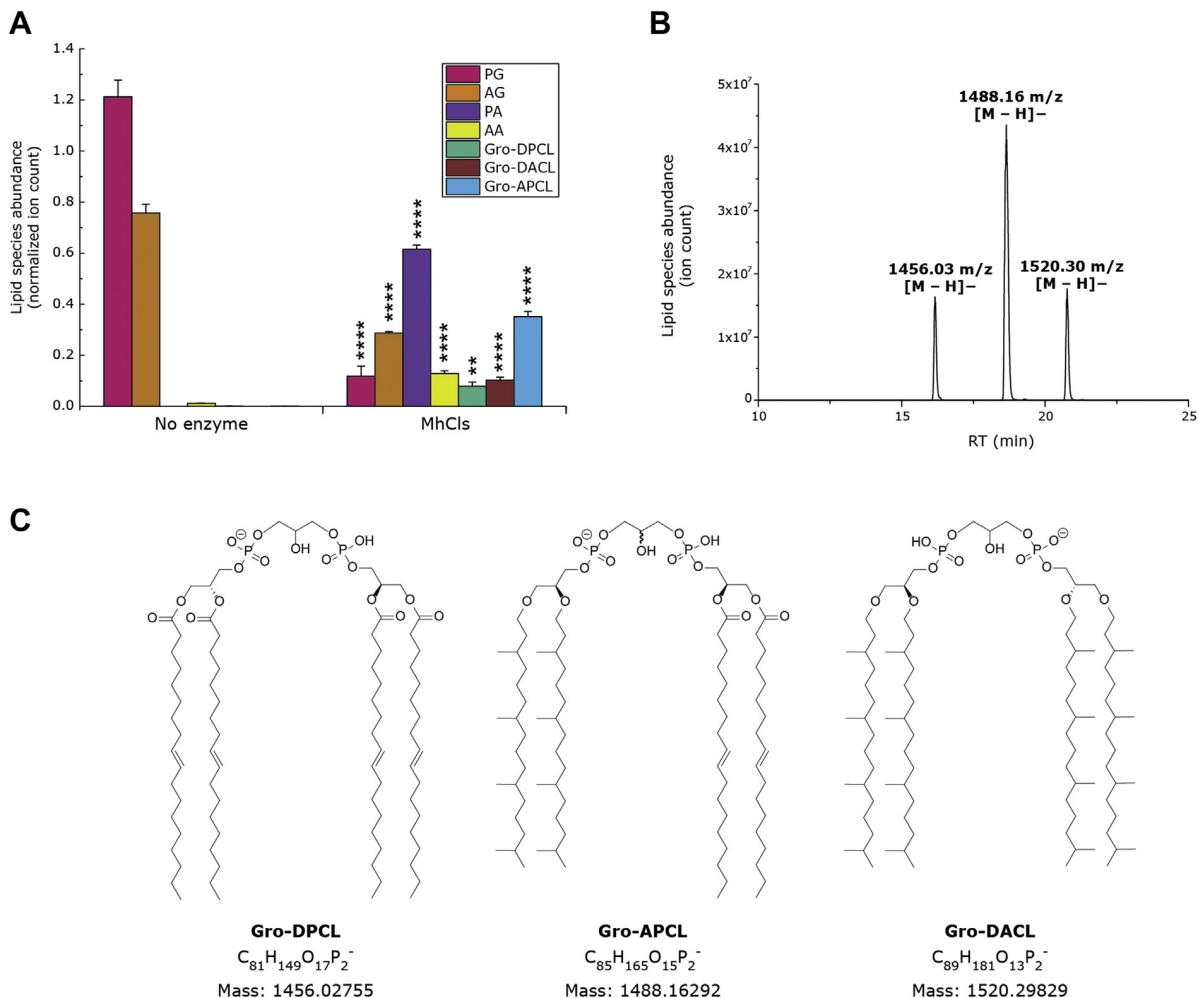
**Synthesis of a bacterial–archaeal hybrid cardiolipin species.***A*, activity of the archaeal MhCls in the presence of both archaetidylglycerol (AG) and phoshpatidylglycerol (PG). Lipid species PG, AG, phosphatidic acid (PA), archaetidic acid (AA), glycerol-di-phosphatidyl-cardiolipin (Gro-DPCL), glycerol-archaetidyl-phosphatidyl-cardiolipin (Gro-APCL), and glycerol-di-archaetidyl-cardiolipin (Gro-DACL) were analyzed by LC-MS, normalized for the internal standard, and plotted. Data are mean ± SD (n = 3). Statistical significance is shown for the enzymatic reaction (MhCls) compared with the control (no enzyme), for each individual lipid species, by using the Student’s *t*-test analysis; ∗∗*p* ≤ 0.01; ∗∗∗∗*p* ≤ 0.0001. *B*, LC-MS chromatogram showing the separation of the produced bacterial Gro-DPCL, hybrid Gro-APCL, and archaeal Gro-DACL. *C*, structures of the three cardiolipin species. Note that in Gro-APCL, the presence of the archaetidyl-group and the phosphatidyl-group makes the central carbon atom of the glycerol head group a chiral center.

Like most archaeal lipids, AG consists of two isoprenoid chains that are coupled to a glycerol-1-phosphate (G1P) backbone *via* an ether bond, whereas PG, present in Bacteria and Eukaryotes, exists as fatty acid tails that are ester-linked to a glycerol-3-phosphate (G3P). This makes that the glycerol backbone of the archaeal AG has the opposite chirality compared with bacterial/eukaryotic PG. To further examine the lipid specificity of MhCls, its activity was examined in the presence of a mixture containing both PG and AG (Fig. 5*A*). Remarkably, not only Gro-DPCL and Gro-DACL were produced, but an additional cardiolipin species was detected: *m/z* 1488.16 [M-H]^−^, which contains one set of isoprenoid lipid tails and one set of fatty acid lipid tails (Fig. 5, *B* and *C*; for fragmentation data see Fig. S7). This lipid species represents a unique archaeal–bacterial hybrid glycerol-archaetidyl-phosphatidyl-cardiolipin (Gro-APCL). Moreover, Gro-APCL seems to be the major produced CL species, while about the same amount of ions are detected for Gro-DACL and Gro-DPCL, suggesting that there is no clear preference for any of the lipid substrates PG or AG.

### Diverse polar headgroup incorporation

**Figure 6:**
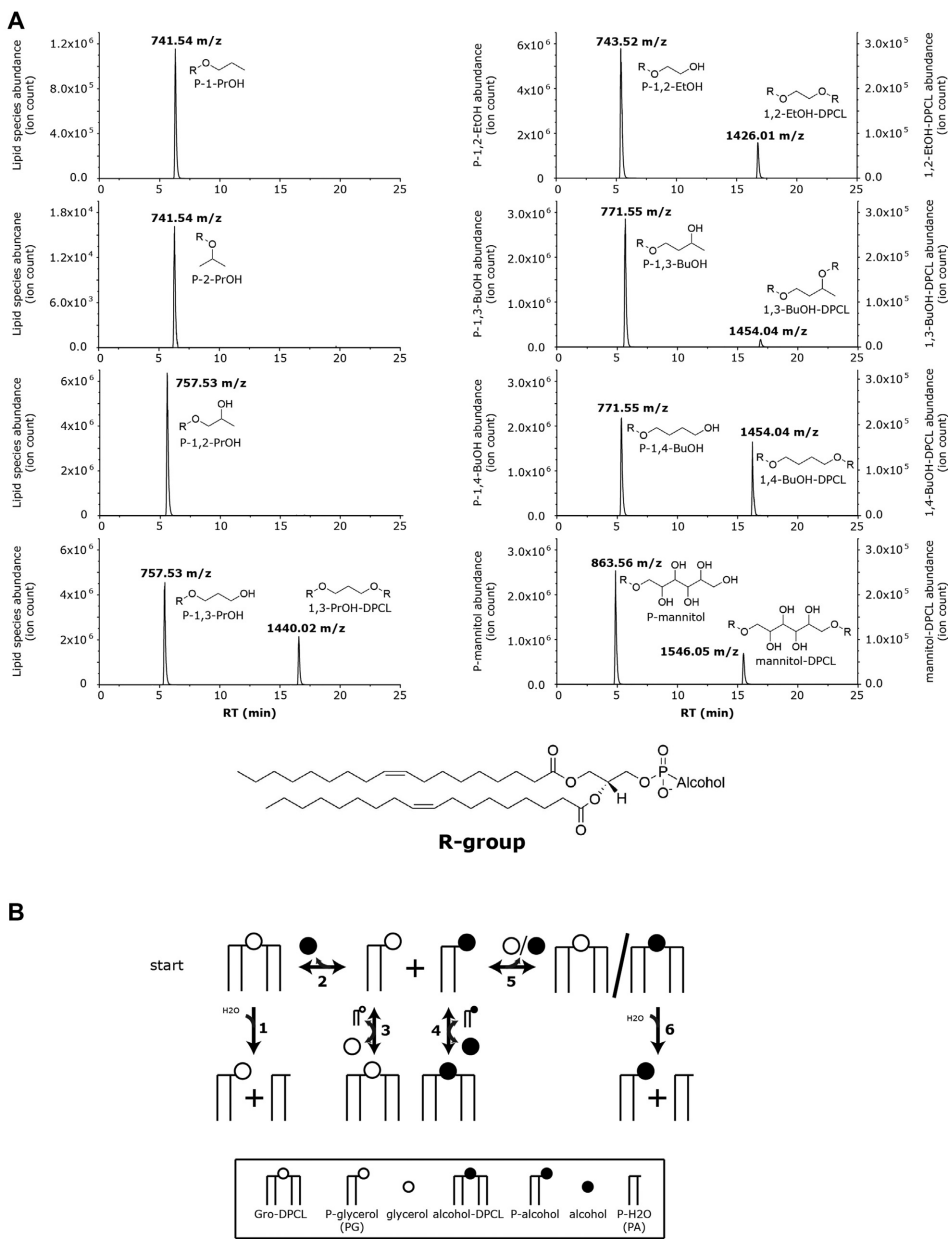
**MhCls activity in the presence of various alcohols I.***A*, glycerol-like alcohols: 1-propanol (1-PrOH), 2-propanol (2-PrOH), 1,2-propanediol (1,2-PrOH), 1,3-propanediol (1,3-PrOH), 1,2-ethanediol (1,2-EtOH), 1,3-butanediol (1,3-BuOH), 1,4-butanediol (1,4-BuOH), and mannitol. Lipid species are displayed as LC-MS chromatograms. *B*, schematic representation of all possible enzymatic reactions performed and products formed by MhCls, starting with glycerol-di-phosphatidyl-cardiolipin (Gro-DPCL) and a free alcohol containing two primary hydroxyl groups as substrates (*left top*). Reaction 1 and 6: hydrolysis of Gro-DPCL and alcohol-DPCL, respectively. Reactions 2, 3, 4, and 5: reversible transesterification of Gro-DPCL/alcohol-DPCL in the presence of glycerol/alcohol into P-alcohol/P-glycerol.

In the MhCls-mediated conversion of Gro-DPCL, either glycerol or H_2_O is utilized. This raises the question: which other molecules can be used by this enzyme? Therefore, molecules structurally related to glycerol were tested in the Gro-DPCL transesterification reaction (Fig. 6*A*). In the presence of 1-propanol, Gro-DPCL consumption resulted in the production of substantial amounts of phosphatidyl-1-propanol (P-1-PrOH). On the other hand, the addition of 2-propanol resulted only in low levels (∼1%) of phosphatidyl-2-propanol (P-2-PrOH), illustrating the importance of a primary hydroxyl (–OH) group for the transesterification. Similar to 1-propanol, 1,2-propanediol and 1,3-propanediol functioned together with Gro-DPCL as substrates for MhCls, resulting in the formation of phosphatidyl-1,2-propanol (P-1,2-PrOH) and phosphatidyl-1,3-propanol (P-1,3-PrOH), respectively. However, in the case of 1,3-PrOH, a clear additional ion *m/z* 1440.20 [M-H]^−^ could be identified, which corresponds to the molecule 1,3-propanediol-di-phosphatidyl-cardiolipin (1,3-PrOH-DPCL), a Gro-DPCL analogue containing a propanediol head group instead of a glycerol. This remarkable cardiolipin species could have only been formed if P-1,3-PrOH functioned as a phosphatidyl-acceptor instead of PG in the cardiolipin forming reaction.

As in the presence of 1,2-propanol, only trace amounts of 1,2-PrOH-DPCL could be detected, it seems that a second primary hydroxyl group is essential for the formation of cardiolipins. This was further tested with the substrates 1,2-ethanediol, 1,3-butanediol, and 1,4-butanediol. All three molecules functioned as a substrate for MhCls in the Gro-DPCL consuming reaction, which resulted in the production of the respective diester phospholipids, P-1,2-ethanediol (P-1,2-EtOH), P-1,3-butanediol (P-1,3-BuOH), and P-1,4-butanediol (P-1,4-BuOH). Moreover, for all these reaction conditions, a cardiolipin equivalent could be detected as well. However, in the presence of 1,3-butanediol, instead of 1,4-butanediol, only 15% of butanediol-di-phosphatidyl-cardiolipin (BuOH-DPCL) could be produced, indicating that a primary hydroxyl group is preferred over a secondary hydroxyl group as a phosphatidyl acceptor. This was further confirmed by the incorporation of mannitol (a six-carbon polyol), which resulted in the production of both phosphatidyl-mannitol (P-mannitol) and mannitol-di-phosphatidyl-cardiolipin (mannitol-DPCL), respectively. These experiments do not only show that the enzyme can utilize a wide variety of primary alcohols, but also exemplify its versatility. By starting with an isomerically pure, symmetric, DPCL species and a substrate containing two primary hydroxyl groups, up to four additional lipid species can be synthesized (Fig. 6*B*). The number of species can be further increased by using an isomerically pure, asymmetric DPCL-species instead, in which also variations in the acyl chain configuration contribute.

**Figure 7:**
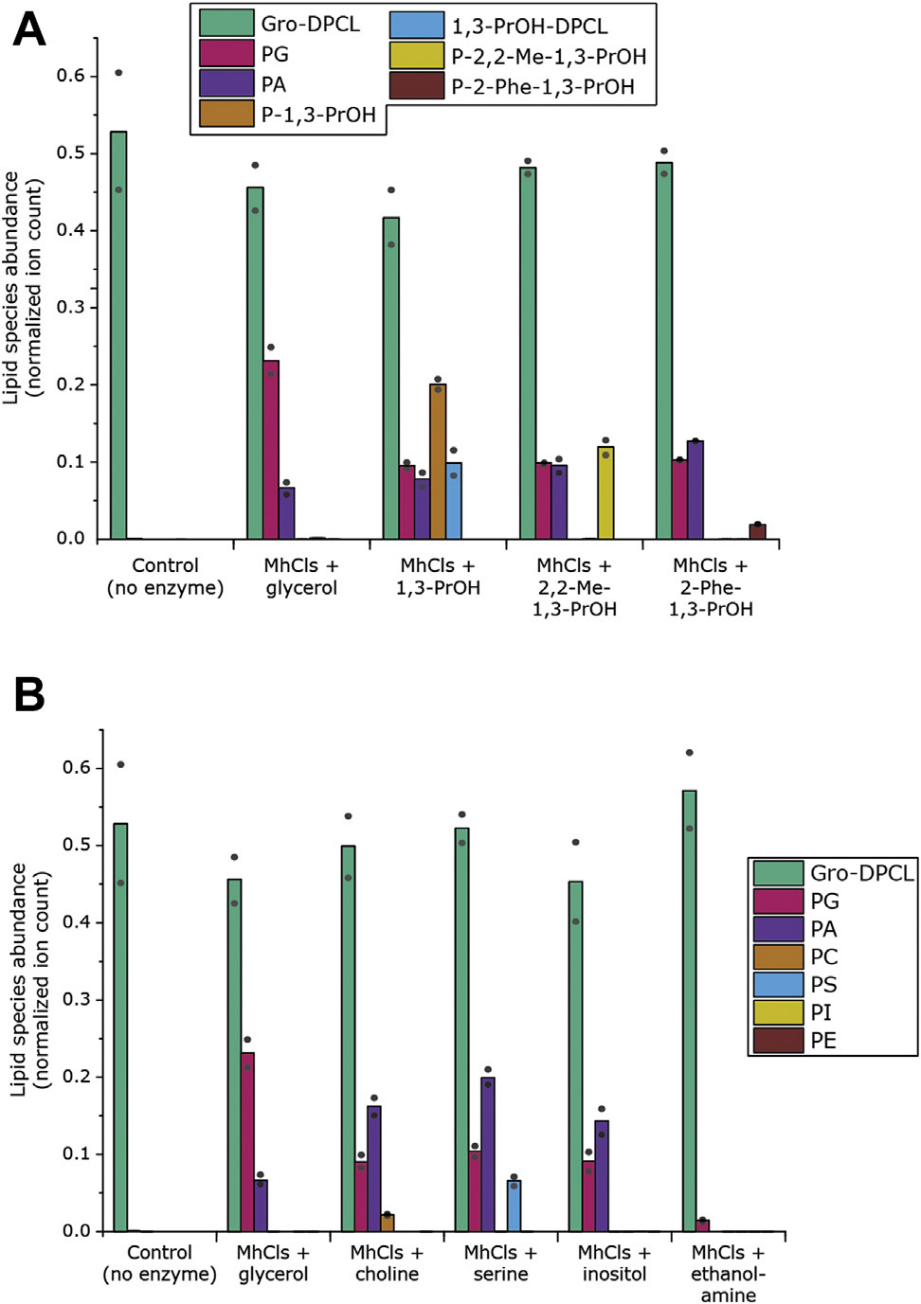
**MhCls activity in the presence of various alcohols II.***A*, 1,3-Propanediol (1,3-PrOH) and its derivatives: glycerol (1,2,3-propanetriol), 2,2-dimethyl-1,3-propanediol (2,2-Me-1,3-ProH) and 2-phenyl-1,3-propanediol (2-Phe-1,3-PrOH). *B*, common polar lipid headgroup alcohols: choline, serine, inositol, ethanolamine. The lipid species glycerol-di-phosphatidyl-cardiolipin (Gro-DPCL), phosphatidylglycerol (PG), phosphatidic acid (PA), and all other phosphatidyl-alcohol (P-alcohol) lipid species were analyzed by LC-MS, normalized for the internal standard DDM, and plotted. Bars represent the mean of the data points (n = 2).

Next, we introduced molecules similar to glycerol, but with different bulky side chains at the C-2 atom (Fig. 7*A*). The presence of two methyl groups or a phenyl group at the C-2 position of the propanediol did not prevent these compounds from being used as a substrate, which resulted in the production of phosphatidyl-2,2-dimethyl-1,3-propanediol (P-2,2-Me-1,3-PrOH) and phosphatidyl-2-phenyl-1,3-propanediol (P-2-Phe-1,3-PrOH). However, only trace amounts of the respective cardiolipin equivalents could be formed, which indicates that these phosphatidyl-alcohol lipids do not function as suitable phosphatidyl acceptors, possibly because of the steric hindrance of the substituents on the lipid headgroup.

Furthermore, a series of primary alcohols of biological relevance were tested. Herein, choline, L-serine, and ethanolamine were included in the reaction. In the case of choline and L-serine, the phospholipid species phosphatidylcholine (PC) and phosphatidylserine (PS) were formed together with PG and PA (Fig. 7*B*). However, in the presence of ethanolamine, no phosphatidylethanolamine (PE) was formed. Notably, the production of PA and PG was largely abolished as well, suggesting that ethanolamine is an inhibitor of MhCls. An inhibiting effect was also observed in the presence of 3-amino-propanol (Fig. S10*A*). Likewise, MhCls is unable to synthesize Gro-DPCL or PA from a reaction mixture of PG in the presence of either 3-amino-propanol or ethanolamine, suggesting that primary amines inhibit the enzyme (Fig. S10*B*). Finally, the sugar inositol was also tested even though this molecule does not contain any primary hydroxyl groups, and as expected no phosphatidylinositol (PI) was found.

### Glycocardiolipin formation

**Figure 8:**
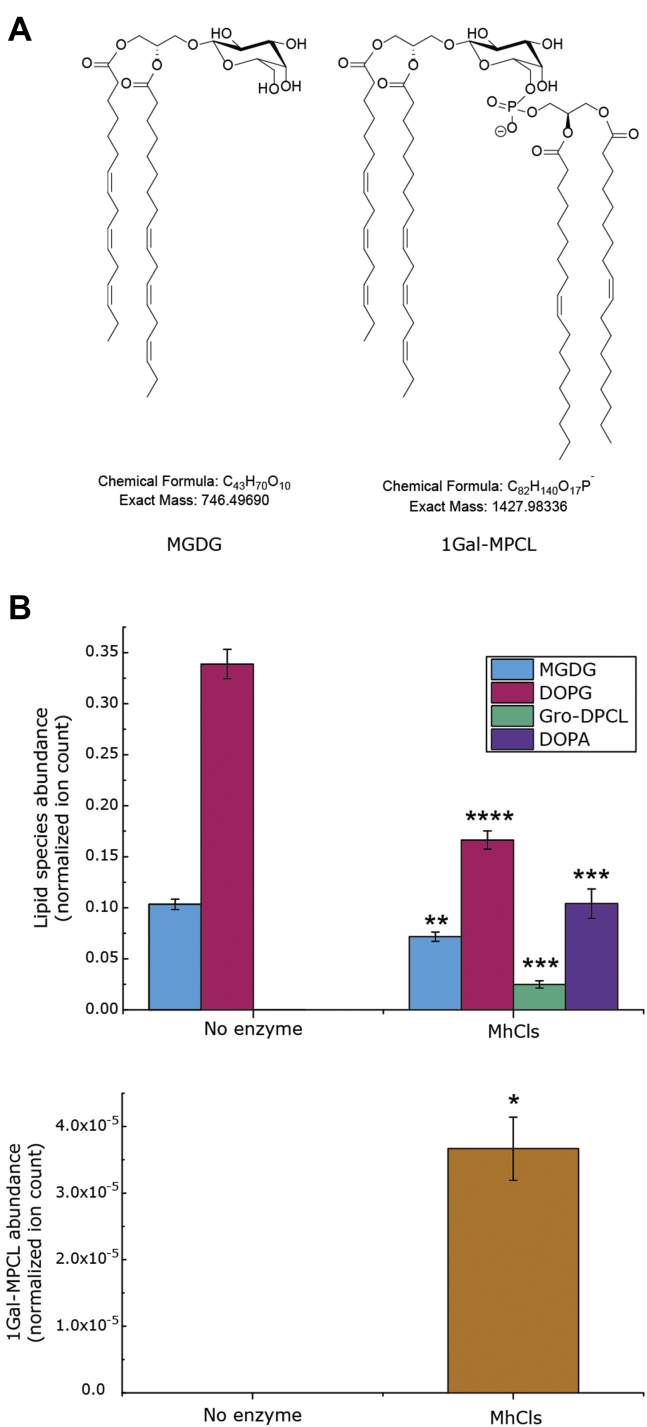
**MhCls-dependent glyco-mono-phosphatidyl-cardiolipin formation.***A*, structures of glycolipid mono-galactosyl-diacylglycerol (MGDG) and 1-galactosyl-mono-phosphatidyl-cardiolipin (1Gal-MPCL). *B*, MhCls-mediated formation of glycerol-di-phosphatidyl-cardiolipin (Gro-DPCL), di-oleoyl-phosphatidic acid (DOPA), and 1Gal-MPCL from the substrates MGDG and di-oleoyl-phosphatidylglycerol (PG). Lipid species were analyzed by LC-MS, normalized for the internal standard DDM, and plotted. Data are mean ± SD (n = 3). Statistical significance is shown for the enzymatic reaction (MhCls) compared with the control (no enzyme), for each individual lipid species, by using the Student’s *t*-test analysis; ∗*p* ≤ 0.05; ∗∗*p* ≤ 0.01; ∗∗∗*p* ≤ 0.001; ∗∗∗∗*p* ≤ 0.0001.

Since archaea also contain glycosyl-mono-archaetidyl-cardiolipin (glyco-MACL) species, the question arises if MhCls could also catalyze their synthesis (19). These molecules basically consist of a glycolipid ester-bonded to a phospholipid, thus containing only one instead of two phosphate moieties and a sugar headgroup instead of a glycerol (Fig. 8*A*). To examine the ability of MhCls to make a glyco-MACL/MPCL, monogalactosyldiacylglycerol (MGDG), a glycolipid species present in the thylakoid membrane of higher plant chloroplasts, was tested as a possible substrate together with PG. Noteworthy, the MGDG used is a mixture of natural lipids with different fatty acid compositions. For simplicity we focused on the most abundant MGDG species (65–70%) with the acyl-chain configuration: 16:3 to 18:3 (*m/z* 745.49 [M-H]^−^). To promote glyco-MPCL formation over Gro-DPCL, MGDG was added in a twofold excess compared with PG. However, since MGDG is a nonbilayer forming lipid, an excess of PC was also added to ensure bilayer formation for enzyme reconstitution, which resulted in a molar PG:MGDG:PC ratio of 1:2:17. In the presence of MhCls, the expected products, Gro-DPCL and PA were formed in substantial amounts with concomitant utilization of PG (Fig. 8*B*). Furthermore, small amounts of another compound (*m/z* 1427.98 [M-H]^−^, mass error: 0.91 ppm) could be detected, corresponding to the glyco-MPCL species mono-galactosyl-mono-phosphatidyl-cardiolipin (1Gal-MPCL) (Fig. 8*B*). Although the signal is low, it is clearly detectable and elutes during the expected retention time range. MGDG elutes earlier from the column compared with PG, and therefore it is expected that the retention time of 1Gal-MPCL is also shorter than that of Gro-DPCL (Fig. S11). Moreover, this mass is not present in the control condition without enzyme (Fig. 8*B*) or without MGDG (data not shown), confirming that this molecule can only be formed by MhCls in the presence of MGDG.

## Discussion

Cardiolipins are lipid species found in the membranes of all domains of life. In contrast to Eukaryotes and Bacteria, the enzymes responsible for cardiolipin biosynthesis have not been studied in Archaea. Here, we identified the cardiolipin synthase from *M. hungatei* (MhCls), which is a member of the phospholipase D superfamily (33), and characterized its function. Archaeal phospholipids consist of isoprenoid tails that are ether-linked to a glycerol-1-phosphate backbone. As a result, their chemical composition and chirality differ from the bacterial and eukaryotic lipids made of fatty acid tails that are coupled to a glycerol-3-phosphate backbone *via* an ester bond. Nevertheless, MhCls indiscriminately uses AG or PG to generate Gro-DACL and Gro-DPCL, respectively. This promiscuous feature of the enzyme enables it to simultaneously utilize PG and AG, resulting in the production of a novel archaeal–bacterial hybrid-cardiolipin species, Gro-APCL, which contains one archaetidyl- and one phosphatidyl-moiety bridged by a glycerol headgroup. So far, such a molecule has not been observed in natural membranes, which can be attributed to an evolutionary event in which our last universal common ancestor (LUCA) evolved into the domains of Archaea and Bacteria. This event is also known as the “lipid divide” and distinguishes both domains with respect to the chemical composition and chirality of their membrane phospholipids (34, 35). The ability of MhCls to synthesize Gro-DACL, Gro-DPCL, and Gro-APCL implies that critical substrate recognition only involves the polar headgroup of AG and PG (36, 37). Our data further shows that MhCls can substitute for the cardiolipin synthases in cardiolipin synthesis when expressed in the *E. coli clsABC* null strain. This demonstrates that the enzyme also has the predicted activity *in vivo*. Preferentially, the role of MhCls should be tested in *M. hungatei*, but for this strict anaerobic organism, there are no genetic tools that enable gene inactivation.

During the *in vitro* formation of these cardiolipin species, substantial levels of archaetidic acid (AA) and phosphatidic acid (PA) were noted, which emerged from the hydrolytic degradation of Gro-DACL and Gro-DPCL, respectively. As a consequence, cardiolipin synthases may act in lipid remodeling. For instance, the PA produced through the hydrolysis of Gro-DPCL can be reutilized by the enzyme CDP-diacylglycerol synthase (CdsA), for the formation of other phospholipid species (38, 39). The same accounts for the produced AA, which can be recycled back into the lipid biosynthesis route by CDP-archaeol synthase CarS (40). However, *in vitro* we show that in the presence of only MhCls, PA cannot be reutilized and accumulates in time, eventually depleting the PG-Gro-DPCL pool.

Our data show that MhCls catalyzes a glycerol-dependent dynamic equilibrium between its product Gro-DPCL and the substrate PG. However, the enzyme exhibits a remarkable substrate promiscuity toward the lipid head group. Besides glycerol (and H_2_O), MhCls can incorporate various other substrates in the cardiolipin-utilizing reaction, yielding a wide variety of phosphatidyl-containing lipid species. Various primary alcohols can be attached to a phosphatidyl group in the Gro-DPCL utilizing reaction, which results in the formation of PG and the specific phosphatidyl-alcohol. This is illustrated by the ability of MhCls to incorporate ethanediol, a two-carbon-diol, as well as the six-carbon-polyol mannitol, showing the enzyme its flexibility toward the length of the carbon chain. Moreover, 1,3-propanediol derivatives with varying bulky side groups at the second carbon can serve as phospholipid head group as well. Besides that, compounds with more biological relevance were tested, in which PC and PS could be formed during the conversion of Gro-DPCL in the presence of choline and serine, respectively. However, substrates that contain a primary amine (*e.g.*, ethanolamine) act as inhibitors of the enzyme in both the cardiolipin biosynthetic and hydrolytic reaction. In addition, some phosphatidyl-alcohol species that are synthesized from a substrate that contains a second primary hydroxyl group can be further converted into a Gro-DPCL analogue with the alcohol as bridging head group, thereby forming atypical and nonnatural cardiolipin species. The versatility of MhCls is further exemplified by the production of the glycosyl-mono-phosphatidyl-cardiolipin 1Gal-MPCL from the glycolipid substrate MGDG and the phospholipid substrate PG. This molecule was only detected in small amounts, indicating that MGDG (originating from plants) is a poor substrate, but the data support the notion that this enzyme can produce a glycocardiolipin and that their synthesis might not involve a separate class of enzymes. Thus in halophiles, the identified glycosyl-mono-archaetidyl-cardiolipin species likely arise from a reaction that involves archaetidylglycerol and the respective glycolipid precursor (20, 21, 22).

Our *in vitro* assays show that MhCls can perform a wide variety of catalytic reactions. They are all based on the reversible transfer of a primary alcohol to a phosphatidyl group. The implications of this promiscuity for the biological function of MhCls are as yet unclear. So far, no cardiolipin species have been reported in the *M. hungatei* lipidome (41, 42), but those lipidomics studies were performed under growth conditions where the *MhCls* gene is barely expressed (43). This is not an uncommon finding as in many bacteria and, insofar studied, archaea, the cardiolipin synthases are predominantly expressed during specific conditions (*e.g.*, osmotic shock, specific growth phase, etc.), yielding different levels of cardiolipins (15, 16, 17, 18). In this respect, the observed reversibility of the phosphatidyl-transfer makes the abundance of cardiolipins flexible, which could aid in the environmental response. Moreover, the adaptive ability of the membrane might be diversified with the promiscuous behavior of MhCls, illustrated by the ability of the enzyme to accept a wide variety of primary alcohols and lipids, which could be a general feature for cardiolipin synthases (27, 44, 45, 46).

Finally, the promiscuity of MhCls could be utilized for bioengineering purposes. As an example, the ability of this enzyme to incorporate a wide variety of non-natural polar head groups into phospholipid species may lead to new bio-catalytic applications for the synthesis of unique phospholipid species. Furthermore, MhCls potentially could be used for the bottom-up construction of a synthetic cellular membrane, in which this enzyme could diversify the phospholipid head group composition of an expanding phospholipid bilayer (38).

## Experimental procedures

### Bioinformatic identification of MhCls

Using *E. coli* K12; MG1655 ClsA (EcClsA: NP_415765.1), ClsB (EcClsB: WP_187790083), or ClsC (EcClsC: WP_188006884.1) as query sequences, BLAST homology searches to the domain of Archaea were performed with the following result: EcClsA: query coverage 75 to 98% and sequence identity 23 to 31%; EcClsB: query coverage 76 to 88% and sequence identity 25 to 33%; EcClsC: query coverage 69 to 91% and sequence identity 21 to 28%. The BLAST results were further analyzed using MEGA X and filtered for sequences that contain at least two HKD domains. Next, sequences were aligned using the MUSCLE algorithm (default settings) and a phylogenetic tree was estimated using the LG+G model (47). Subsequently, a putative archaeal cardiolipin synthase (MhCls: WP_011448254) from *M. hungatei* JF-1 was selected. The consensus sequence logo was created with the program WebLogo (https://weblogo.berkeley.edu/logo.cgi), for which the group of methanogens was selected together with a group of bacterial EcClsA homologs (see Supporting information). This resulted in a group of 90 species (57 bacteria and 33 archaea), from which one archaeal sequence, containing many additional amino acids within the second hydrophobic domain, was removed.

### Bacterial strains and cloning procedures

An *E. coli* codon-optimized synthetic gene of *M. hungatei* Cls (MhCls) was ordered (GeneArt, Thermo Fisher scientific) and used as a template for the amplification of MhCls, during which an N-terminal 6xHis-tag was added. The 6His-MhCls fragment was cloned into a pRSF-Duet expression vector, using the NcoI and SacI restriction enzymes and T4 DNA ligase, resulting in pRSF-6His-MhCls (pNDK001). *E. coli* DH5α (Invitrogen) was used as a host for Cloning procedures. *E. coli* Lemo21 (DE3) was used as the overexpression strain for pNDK001. The 6His-MhCls fragment was also cloned into a pBAD expression vector, using the EcoRI and HindIII restriction enzymes and T4 DNA ligase, resulting in pBAD-6his-MhCls (pME006). *E. coli* DH5α (Invitrogen) was used as a host for Cloning procedures. *E. coli clsABC* null strain (K12 Δ*clsA*, Δ*clsB*, Δ*clsC*::*kanR* (BKT12)) (17) was used as the overexpression strain for pME006. All primers and plasmids used in the present study are listed in Tables 1 and 2. All strains were grown under aerobic conditions at 37 °C in LB medium supplemented with the required antibiotics: kanamycin (50 μg/ml), chloramphenicol (34 μg/ml), Zeocin (25 μg/ml).

**Table 1:** Cloning and expression vectors used in this study

Plasmid	Description	Reference
pRSF-Duet-1	Expression vector (Kan^R^), T7 promoter	Novagen
pNDK001	*MhCls* gene with N-terminus 6x His-tag from *M. hungatei JF-1* cloned into pRSF-Duet vector using the primers NDKo027 and NDKo028.	This study
pBAD	Expression vector, pBR322 ori; araC; pBAD, Zeo^R^	This study
pME006	*MhCls* gene with N-terminus 6x His-tag from *M. hungatei JF-1* cloned into pBAD-vector using the primers prME010 and prME011 to amplify the *MhCls* gene, and primers prME012 and prME013 to amplify the pBAD backbone.	This study

**Table 2:** Oligonucleotide primers used in this study

Primers	Primer sequence 5’ -> 3’	Restriction site
NDKo027	ACAGTTCCATGGCCCATCACCATCATCACCACATCCATGATCTGATTCTGGTGATCCACAATTTTC	NcoI
NDKo028	ACTTACGAGCTCTTATTATTACAGCAGCGGACTAAACAGACG	SacI
prME010	TATCGAATTCATGCATCACCATCATCACCACATCCATGATCTGA	EcoRI
prME011	CTATAAGCTTTTATTATTACAGCAGCGGACTAAACAGACGGCTAAT	HindIII
prME012	ACTCGAATTCTTCCTCCTAGCCTGCTTTTTTGT	EcoRI
prME013	ACTGAAGCTTGATATCGTTTAAACGGTCTCCAGCTTGG	HindIII

Introduced restriction sites are underlined.

### Expression and purification of MhCls

MhCls was overexpressed in *E. coli* Lemo21 (DE3) strain in the presence of 250 μM rhamnose and induced with 0.5 mM isopropyl β-D-1-thiogalactopyranoside (IPTG). After 2.5 h of induction, cytoplasmic and membrane fractions were separated as described (48). The total membranes were resuspended in buffer A (50 mM Tris/HCl, pH 8.0, 100 mM KCl and 15% glycerol) after which they could be stored at −80 °C. For further purification, 0.5 mg/ml of membranes was solubilized in 2% n-dodecyl-β-D-maltopyranoside (DDM) detergent for 1 h at 4 °C. The material was subjected to a centrifugation (17,000*g*) step for 15 min at 4 °C to remove insolubilized material and the supernatant was incubated with Ni-NTA agarose beads (Qiagen, cat: 30230) for 2 h at 4 °C. The Ni-NTA beads were washed ten times with six column volumes (CV) of buffer B (50 mM Tris/HCl, pH 8.0, 100 mM KCl, 15% glycerol and 0.05% DDM) supplemented with 10 mM imidazole, and the protein was eluted three times with 0.5 CV of buffer B supplemented with 300 mM imidazole. To remove the imidazole and glycerol, the purified protein was passed over a Zeba Spin Desalting column 40K; 0.5 ml (Thermo scientific) and eluted in buffer C (50 mM MES pH 7.0, 100 mM KCl and 0.05% DDM). Purity of the eluted protein was assessed on 15% SDS/PAGE stained with Coomassie Brilliant Blue, and the protein concentration was determined by measuring the absorbance at 280 nm and calculating the molar concentration using the calculated extinction coefficient. Extinction coefficients were obtained from the ProtParam tool from the ExPASy website (https://web.expasy.org/protparam/).

### Total lipid extraction

Lipids were extracted from 10 mg freeze-dried cells from *E. coli clsABC* null strain pellets using an adapted Bligh and Dyer method employing 5% trichloroacetic acid as described elsewhere (49). The crude chloroform fraction was dried and the lipid film was re-extracted with 400 μl chloroform– methanol (1:2), dried again, and finally reextracted with 50 μl methanol.

### Liposomes preparation

Chloroform stocks of the lipid species DOPG, POPG, DOPA, POPA, DOPE, DOPC, Gro-DPCL 18:1/18:1/18:1/18:1, and MGDG were purchased from Avanti (Avanti Polar Lipids). The chemical synthesis of AG was performed in house and is described in detail in the Supporting information. For liposomes with a heterogeneous lipid mixture, the required amount of lipid chloroform stocks was mixed together in the stated molar ratio. Next the lipid solution was dried under a nitrogen gas stream for multiple hours, after which the dry lipid film was resuspended in a 50 mM 2-(N-morpholino)ethanesulfonic acid (MES) buffer, pH 7.0 yielding a translucent suspension. For formation of liposomes, a probe sonicator was employed (30 s cycle time with a 50% duty cycle for 10–20 cycles) until the suspension became transparent.

### *In vitro* assays for phospholipid synthesis

All *in vitro* reactions were performed in 100 μl of buffer D containing a final concentration of 50 mM MES pH 7.0 and 100 mM KCl in the presence of 1 μM MhCls. The activity of MhCls with archaeal substrate was assayed in the presence of 250 μM AG (*sn*1-*sn*1 and *sn*1-*sn*3; ratio 1:1) and 0.4 mM DDM. The glycerol-dependent dynamic equilibrium of MhCls was assayed in the presence of 1.8 mM DDM and 1 mM DOPG, 0.5 mM Gro-DPCL, or 0.5 mM POPG together with 0.25 mM DPCL. The activity of MhCls with AG and PG (molar ratio 1:1) was assayed with 250 μM of each lipid substrate in the presence of 0.8 mM DDM. The promiscuity toward primary-hydroxyl-containing compounds was assayed with 0.5 mM Gro-DPCL or 1 mM DOPG, 100 mM primary-hydroxyl-containing substrate, and 1.8 mM DDM. Formation of 1Gal-MPCL was performed in the presence of 1 μM MhCls, 0.5 mM lipid (PG:MGDG:PC, molar ratio 1:2:17), and 0.8 mM DDM. All reactions were incubated overnight at 37 °C unless stated differently. Lipids were extracted from the reaction mixtures two times with 0.3 ml of 1-butanol and evaporated under a stream of nitrogen gas and resuspended in 50 μl of methanol for LC-MS analysis.

### LC-MS analysis of lipids

Samples from the *in vitro* reactions were analyzed using an Accela1250 high-performance liquid chromatography (HPLC) system coupled with a heated electrospray ionization–mass spectrometry (HESI-MS) Orbitrap Exactive (Thermo Fisher Scientific). A sample of 5 μl was injected into an ACQUITY UPLC CSH C18 1.7 μm Column, 2.1 × 150 mm (Waters Chromatography Ireland Ltd) operating at 55 °C with a flow rate of 300 μl/min. Separation of the compounds was achieved by a changing gradient of eluent A (5 mM ammonium formate in water/acetonitrile 40:60, v/v) and eluent B (5 mM ammonium formate in acetonitrile/1-butanol, 10:90, v/v). The following linear gradient was applied: 45% eluent B for 2.5 min; a gradient from 45% to 90% eluent B over 19.5 min; holding for 3 min; returning to 45% eluent B in 0.5 min; and holding for 8 min. For the *E. coli clsABC* null strain total lipid extracts, 10 μl was injected and the following linear gradient was applied: 5% eluent B for 2.5 min; a gradient from 5% to 90% eluent B over 36.5 min; holding for 3 min; returning to 5% eluent B in 0.5 min; and holding for 8 min. The column effluent was injected directly into the Exactive ESI-MS Orbitrap operating in negative ion mode. Voltage parameters of 3 kV (spray), −75 V (capillary), −190 V (tube lens), and −46 V (Skimmer voltage) were used. Capillary temperature of 300 °C, sheath gas flow of 60, and auxiliary gas flow of 5 were maintained during the analysis.

Spectral data constituting total ion counts were analyzed using the Thermo Scientific XCalibur processing software by applying the Genesis algorithm-based automated peak area detection and integration. The total ion counts of the extracted lipid products (Table 3) were normalized for DDM (*m/z* 509.3 [M-H]^−^) and plotted on the y-axis as normalized ion count in a bar graph.

**Table 3:** Detected lipid species with LC-MS

Lipid species	*m/z* [M-H]^−^
archaetidylglycerol (AG)	805.66
archaetidic acid (AA)	731.63
glycerol-di-archaetidyl-cardiolipin (Gro-DACL)	1520.30
di-oleoyl phosphatidic acid (DOPA), phosphatidic acid (PA)	699.49
di-oleoyl phosphatidylglycerol (DOPG), phosphatidylglycerol (PG)	773.53
palmitoyl-oleoyl phosphatidic acid (POPA)	673.48
palmitoyl-oleoyl phosphatidylglycerol (POPG)	747.52
di-phosphatidyl-cardiolipin (Gro-DPCL), Gro-DPCL18:1/18:1/18:1/18:1	1456.03
Gro-DPCL16:0/18:1/16:0/18:1	1403.99
Gro-DPCL18:1/18:1/16:0/18:1	1430.01
Gro-archaetidyl-phosphatidyl-cardiolipin (APCL)	1488.16
phosphatidyl-1-propanol (P-1-PrOH), and phosphatidyl-2-propanol (P-2-PrOH)	741.54
phosphatidyl-1,2-propandiol (P-1,2-PrOH), and phosphatidyl-1,3-propandiol (P-1,3-PrOH)	757.53
1,2-propanediol-di-phosphatidyl-cardiolipin (1,2-PrOH-DPCL), and 1,3-propanediol-di-phosphatidyl-cardiolipin (1,3-PrOH-DPCL)	1440.02
phosphatidyl-1,2-ethanol (P-1,2-EtOH)	743.52
1,2-ethanol-di-phosphatidyl-cardiolipin (1,2-EtOH-DPCL)	1426.01
phosphatidyl-1,3-butanediol (P-1,3-BuOH), and phosphatidyl-1,4-butanediol (P-1,4-BuOH)	771.55
1,3-butanediol-di-phosphatidyl-cardiolipin (1,3-BuOH-DPCL), and 1,4-butanediol-di-phosphatidyl-cardiolipin (1,4-BuOH-DPCL)	1454.04
phosphatidyl-mannitol (P-mannitol)	863.56
mannitol-di-phosphatidyl-cardiolipin (mannitol-DPCL)	1546.05
phosphatidyl-2,2-dimethyl-1,3-propanediol (P-2,2-Me-1,3-PrOH)	786.56
phosphatidyl-2-phenyl-1,3-propanediol (P-2-Phe-1,3-PrOH)	833.56
di-oleoyl phosphatidylethanolamine (DOPE)	742.54
phosphatidyl-aminopropanol (P-NH2-PrOH)	756.55
di-oleoyl phosphatidylcholine (DOPC)	830.59
di-oleoyl phosphatidylserine (DOPS)	786.53
di-oleoyl phosphatidylinositol (DOPI)	861.55
monogalactosyldiacylglycerol (MGDG)	746.49
monogalactosyl-mono-phosphatidyl-cardiolipin (1Gal-MPCL)	1427.98

## Data availability

All raw and processed data used for, and described in, this article is stored in the department of Molecular Microbiology at the University of Groningen.

## Supporting information

This article contains supporting information (47, 50, 51, 52, 53, 54, 55, 56, 57, 58).

## Conflict of interest

The authors declare that they have no conflicts of interest with the contents of this article.
