# Supplementary material for: A promiscuous archaeal cardiolipin synthase enables construction of diverse natural and unnatural phospholipids
Source: J Biol Chem. 2021 Apr 22;296:100691. doi: 10.1016/j.jbc.2021.100691 (PMC8141893; doi:10.1016/j.jbc.2021.100691)
Supplement: Supplemental Figures S1–S11 [file mmc1.pdf]

Supporting Information

Additional information on the identification of putative archaeal cardiolipin synthases

Cls_pld homologs

The three Cls enzymes (ClsA, ClsB, and ClsC) present in the bacterium *Escherichia coli* were used as template in individual BLAST searches to identify putative archaeal cardiolipin synthases. For these enzymes hits were selected for EcClsA: query coverage 75-98%, and sequence identity 23-31%; EcClsB: query coverage 76-88%, and sequence identity 25-33%; EcClsC: query coverage 69-91%, and sequence identity 21-28%. As the hits obtained with EcClsA showed the best query coverage and sequence identity, it was decided to only continue with the results from EcClsA. Next, the results were filtered for sequences containing at least two HKD motifs, which is a common feature among Cls_pld enzymes. Additionally, some cardiolipin species from bacteria were added. Subsequently the sequences were aligned and a phylogenetic tree was estimated using the LG+G model (Figure S1) (47).

The putative archaeal cardiolipin synthases are divided into two main clusters. One cluster mostly exists of methanogens, whereas the other cluster predominantly contains halophiles that belong to the class of Haloarchaea (Halobacteria). The latter cluster is further subdivided into two groups. An alignment of *Haloferax volcanii* with *Haloterrigena salina* (each representing one of the two clusters), shows there is a query coverage of 84% in which there is a sequence identity of 44%.
To investigate the identity between the methanogenic and halophilic clusters, an alignment of the putative Cls from *Methanospirillum hungatei* and *H. volcanii* was made. The alignment has a query coverage of only 32% in which there is a sequence identity of 26%, clearly indicating two distinct groups. Noteworthy, the bacterial cardiolipin synthases are all located in the methanogenic cluster, in which *E. coli* ClsA seems to be most distinct. An alignment of *E. coli* ClsA with *M. hungatei* Cls shows a query coverage of 95% in which there is a sequence identity of 27%, whereas an alignment of *E. coli* ClsA with *H volcanii* Cls only shows 11% query coverage containing a sequence identity of 27%. These alignments indicate that the methanogenic cluster of putative archaeal cardiolipin synthases are relatively closely related to the bacterial ClsA type, in contrast to the halophilic cluster.

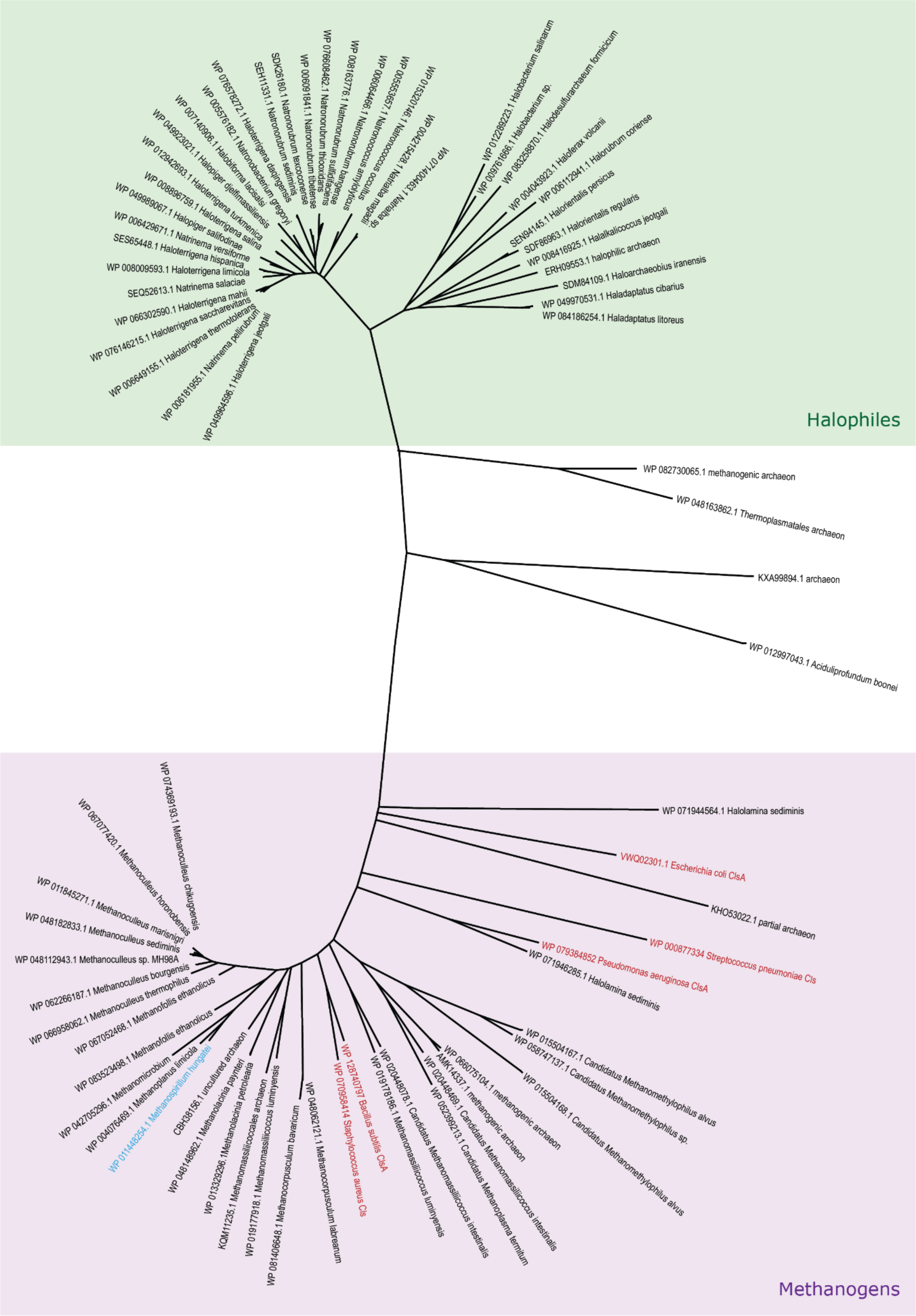

Figure S1: Unrooted tree of putative archaeal cardiolipin synthases that contain two HKD motifs (Cls_pld). Representative bacterial species are highlighted in red and locate in the methanogenic cluster. The putative Cls from *Methanospirillum hungatei* (MhCls) that is characterized in this work is highlighted in blue.

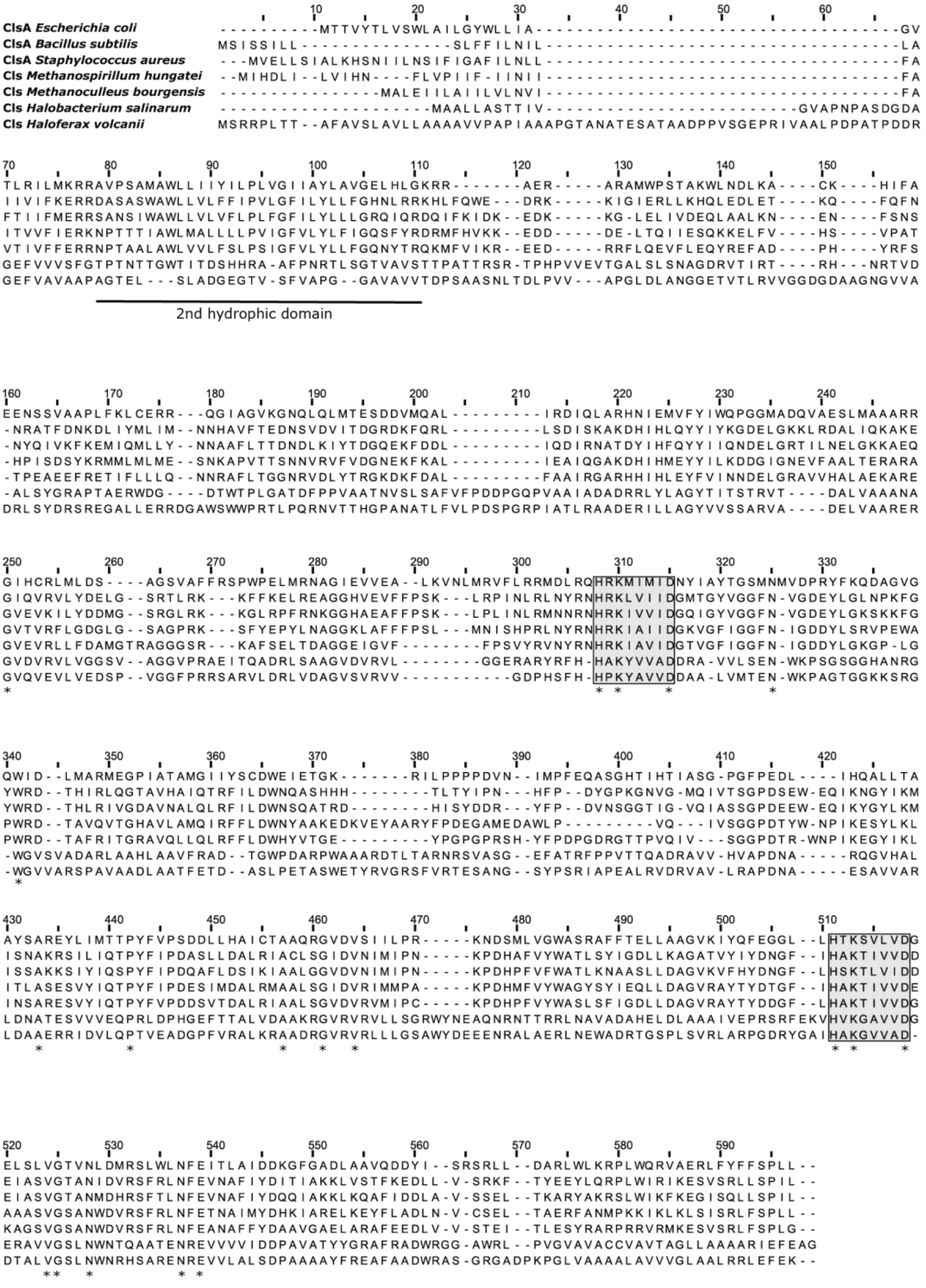

Figure S2: Alignment of *Escherichia coli* ClsA with a selection of bacterial and archaeal (putative) cardiolipin synthases. ClsA from *Escherichia coli* (NP_415765.1) was aligned together with ClsA from *Bacillus subtilis* (WP_128740797), a cardiolipin synthase from *Staphylococcus aureus*, and four putative archaeal cardiolipin synthases from *Methanospirillum hungatei* (WP_011448254), *Methanoculleus bourgensis* (WP_014868452), *Haloferax volcanii* (WP_004043923), *Halobacterium salinarum* (WP_012289223), which represent the methanogenic and halophilic clusters presented in figure 1C. The light grey boxes outline the two conserved HKD motifs. The second hydrophobic domain (Figure 1D) is conserved among the bacterial and methanogenic cardiolipin synthases, but not in the halophiles. The asterisks (*) indicate strictly conserved amino acid positions.

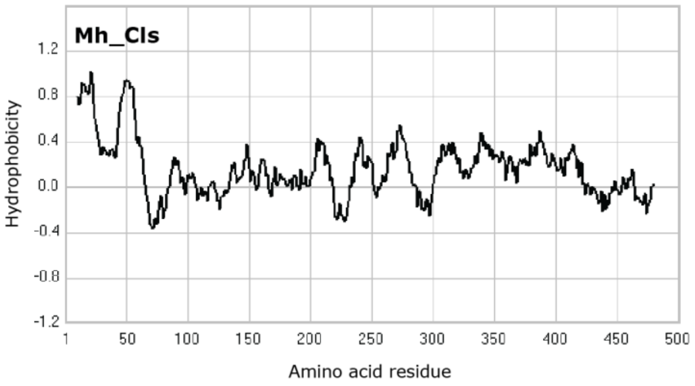

Figure S3: Hydropathy profile of MhCls.

AG synthesis

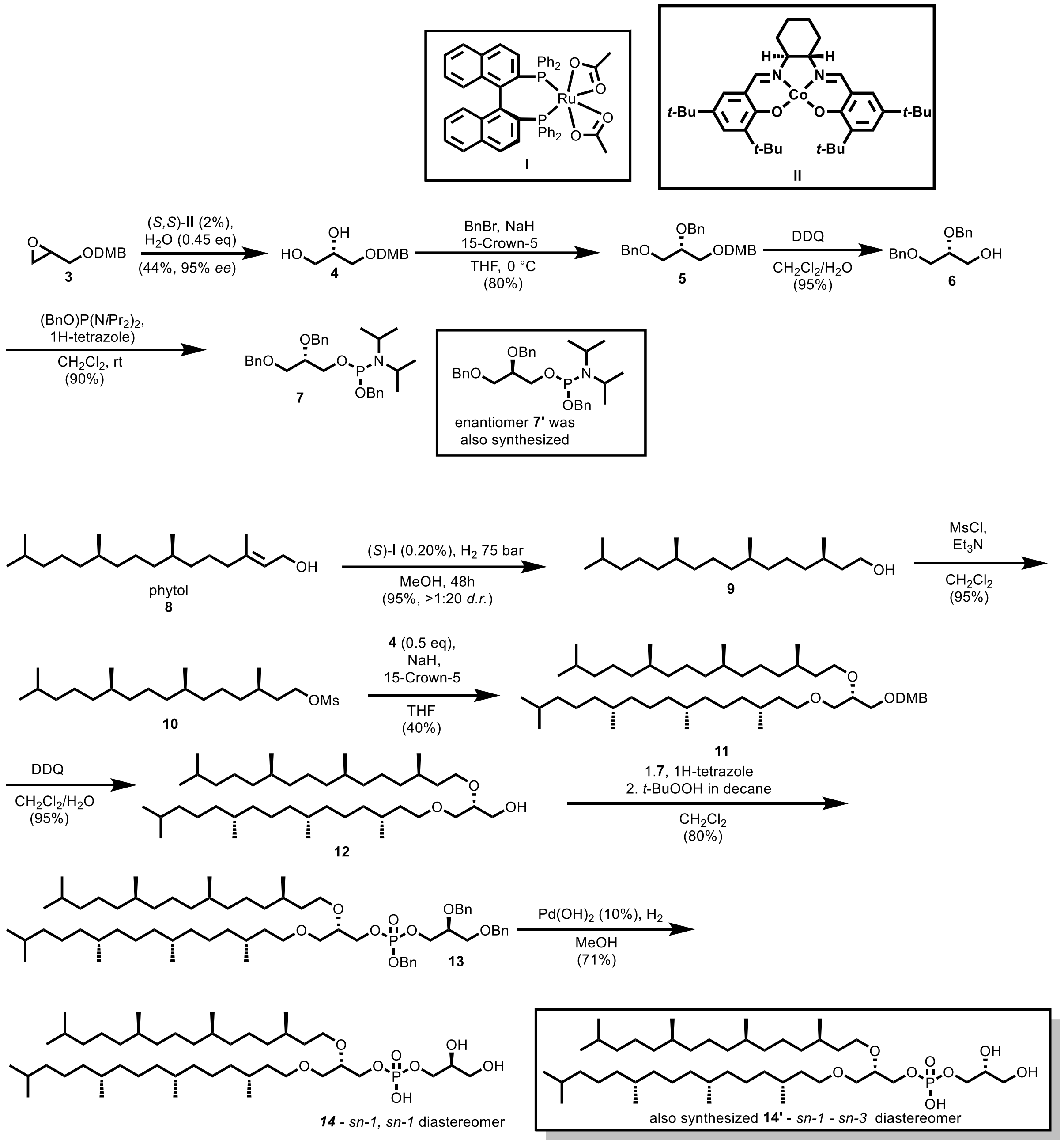

Scheme 1: Total synthesis of the *sn1-sn1* and *sn1-sn3* diastereomers of AG (DDQ: Dicyanodichlorobenzoquinone, 15-C-5: 15-crown-5-ether, DMB: dimethoxybenzyl)

We synthesized both the *sn*1-*sn*1 (**14**) and the *sn*1-*sn*3 (**14’**) diastereomers of AG (Archaetidyl glycerol), as shown in Scheme 1. Both enantiomers of the glycerol headgroup were synthesized from dimethoxybenzyl-protected glycidol via the Jacobsen hydrolytic kinetic resolution, obtaining high yields and *ee*. Benzylation of the two free hydroxy groups followed by deprotection of the DMB-group gave **6** and **6’**, which were converted into their respective benzyloxy-phosphoramidites **7** and **7’**. It should be noted that several attempts were made to prepare **7** with DCI (dicyanoimidazole), a more recently introduced phosphoramidite coupling catalyst, however we observed superior yields with solid 1H-tetrazole.

The phytanyl unit could be readily prepared from commercially available *trans*-phytol, as described by Sita *et al.*(50) It was envisioned that the archaeal diether lipid precursor **12** would be accessible via dialkylation of DMB (dimethoxybenzyl) protected glycerol (**4**). Dialkylation of glycerol derivatives is very challenging due to steric hindrance of the secondary alkoxide, leading to competing elimination of the electrophile. The highest yields were obtained in our hands utilizing the corresponding mesylate and adding crown ether. Smith and co-workers speculate that α-chelating groups make very stable chelates with the alkoxide salt (51). This could explain the increased yield due to disassociation of the chelate when crown ether is added. The DMB group was readily cleaved under oxidative conditions giving the free alcohol in high yield.

The headgroup and lipid fragments were coupled via a similar phosphoramidite coupling, and subsequently oxidized to the phosphate with *t*-BuOOH in one pot. Reductive cleavage of the benzyl groups yielded the desired AGs in good yield.

We were able to determine the selectivity of the ruthenium catalyzed hydrogenation by ^{13}C-NMR-spectroscopy by comparing a diastereomeric mixture of phytanol (*R*,*R*,*R* + *R*,*R*,*S*) with the product of the Noyori asymmetric hydrogenation (*R*,*R*,*R*) which is shown in Figure S4. The characteristic signal of methyl **18** for the *R*,*R*,*R* diastereomer is found at 19.79 ppm and for the *R*,*R*,*S* diastereomer at 19.73 ppm. The signal pattern observed was comparable to that observed in previous studies on 1-5 *syn*/*anti* methyl systems performed by Curran and co-workers (52). Integration of the signals showed a >20:1 selectivity for the all *syn*-methyl-phytanol. Sita observed a similar selectivity by HPLC analysis of chiral carbamate derivatives.

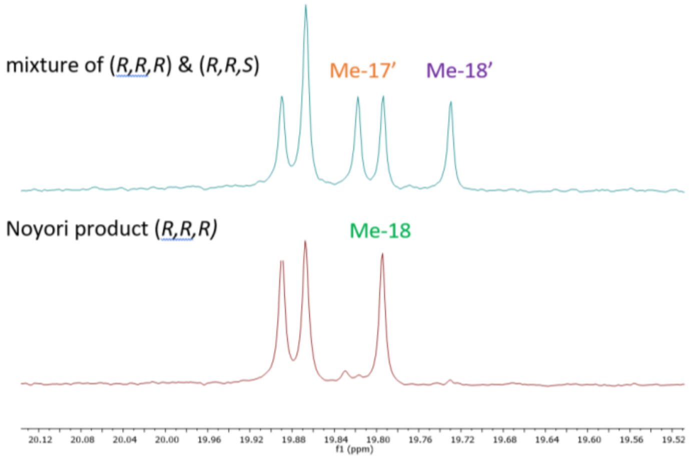

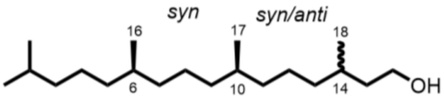

^{13}C-shift	Assignment
19.89	Me-17, *syn*, *syn*
19.87	Me-16, Me-16’, *syn*
19.82	**Me-17’**, *syn*, *anti*
19.79	**Me-18**, *syn*
19.73	**Me-18’**, *anti*

Figure S4: d.r. determination of phytanol via the comparison of the diastereomeric mixture to the enriched sample employing the Curran method (52)**.**

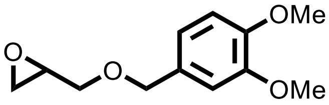

2-(((3,4-dimethoxybenzyl)oxy)methyl)oxirane (3). To a 100 mL 3-necked flask equipped with magnetic stirrer bar was added 25 mL of a 50% NaOH solution, epichlorohydrin (18.5 g, 15.6 mL, 0.2 mol) and Bu_4NHSO_4 (1.5 mmol, 525 mg, 4 mol%). The resulting solution was cooled to 0 ℃ (ice/water-bath) after which neat 3,4-dimethoxybenzyl alcohol (37.5 mmol, 5.5 mL, 6.3 g) was added dropwise over 30 min while the solution was stirred vigorously. The resulting turbid mixture was allowed to warm up to rt over a 5 h period, after which complete conversion was observed by TLC. The entire content of the flask was poured into 100 mL of ice water which was subsequently extracted with diethyl ether (3 x 50 mL). The combined organic layers were washed with brine (2 x 50 mL) dried over $MgSO_4$ and concentrated *in vacuo*. The resulting crude was further purified by column chromatography (1:3 EtOAc/pentane) to give 2-(((3,4-dimethoxybenzyl)oxy)methyl)oxirane as a pale yellow oil (94%, 7.9 g, 35.2 mmol).

^{1}H NMR (400 MHz, Chloroform-*d*) δ 6.94 – 6.78 (m, 3H), 4.52 (q, *J* = 11.6 Hz, 2H), 3.89 (s, 3H), 3,87 (s, 3H), 3.75 (dd, *J* = 11.5, 2.9 Hz, 1H), 3.41 (dd, *J* = 11.4, 5.9 Hz, 1H), 3.19 (td, *J* = 6.3, 3.2 Hz, 1H), 2.80 (t, *J* = 4.6 Hz, 1H), 2.61 (dd, *J* = 4.9, 2.7 Hz, 1H).

^{13}C NMR (101 MHz, Chloroform-*d*) δ 149.0, 148.7, 130.4, 120.4, 111.1, 110.9, 73.2, 70.6, 55.9, 55.8, 50.8, 44.3.

The spectral data correspond to that previously reported (53)

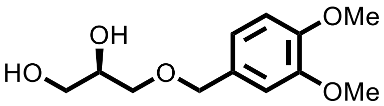

(*S*)-3-((3,4-dimethoxybenzyl)oxy)propane-1,2-diol (4) (54)**.** A 25 mL flask equipped with a magnetic stirrer bar was charged with (*R*,*R*)-**II** (70 mg, 0.005 equiv). The catalyst was exposed to 2-(((3,4-dimethoxybenzyl)oxy)methyl)oxirane (5 g, 22.3 mmol) and AcOH (25 µL, 0.2 equiv). The resulting red mixture was allowed to stir for 30 min in order to oxidize the catalyst. To the resulting brown mixture was added H_2O (220 µL, 0.55 equiv) and was stirred rt for 48 h. The final product was isolated as a brown oil by flash column chromatography (100% EtOAc) (45% yield, 2.2 g, 9.1 mmol).[a] The remaining epoxide (50%) was recovered and could be enriched to 95% *ee* (*R*-enantiomer) with (*S*,*S*)-**II** as catalyst.

Chiral HPLC analysis on a Lux® 5 µm Cellulose-3 column, *n*-heptane : *i*-PrOH = 90 : 10, 40 ˚C, flow = 1 mL/min, UV detection at 274 nm, t_R(major): 25.29 min, t_R(minor): 29.06 min, 97% ee

^{1}H NMR (400 MHz, Chloroform-*d*) δ 6.87 – 6.77 (m, 3H), 4.44 (s, 2H), 3.85 (s, 3H), 3.83 (s, 3H), 3.67 – 3.60 (m, 1H), 3.55 (dd, *J* = 11.5, 5.9 Hz, 1H) 3.52 – 3.42 (m, 2H), 3.03 (br s, 2H).

^{13}C NMR (101 MHz, Chloroform-*d*) δ 149.1, 148.8, 130.3, 120.5, 111.2, 111.0, 73.5, 71.5, 70.8, 64.1, 55.6, 55.9.

$[\alpha]_D^{20}$= +2.4 (c = 0.1 in $CHCl_3$).

---

[a][a] The diol was isolated together with small amounts of the catalyst co-eluting on the column. For optical rotation determination the diol was separated from the catalyst by distillation, yielding a colourless oil. For further reactions the initial column purification was found to be sufficient.

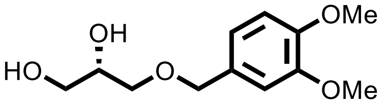

(*R*)-3-((3,4-dimethoxybenzyl)oxy)propane-1,2-diolError! Bookmark not defined. (4'). This compound was prepared with the same synthetic procedure that was used for **(*S*)-3-((3,4-dimethoxybenzyl)oxy)propane-1,2-diol (5)**, using (*S*,*S*)-**II** as catalyst (45% yield).

Chiral HPLC analysis on a Lux® 5 µm Cellulose-3 column, *n*-heptane: *i*-PrOH = 90 : 10, 40 ˚C, flow = 1 mL/min, UV detection at 274 nm, t_R(minor): 26.01 min, t_R(major): 29.21 min, 95% ee.

^{1}H NMR (400 MHz, Chloroform-*d*): Same as reported for compound **4**

^{13}C NMR (101 MHz, Chloroform-*d*): Same as reported for compound **4**

$[\alpha]_D^{20}$= -2.4 (c = 0.1 in $CHCl_3$).

The spectral data correspond to previously reported (55)

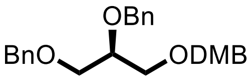

(*R*)-3-((3,4-dimethoxybenzyl)oxy)propane-1,2-di-benzylether (5). Di-methoxybenzyl protected glycerol **4** (500 mg, 2.06 mmol), benzyl bromide (0.736 mL, 6.19 mmol, 3 equiv) and 15-crown-5 (1.4 mL, 7.22 mmol, 3 equiv) were dissolved in THF (4.1 mL) and cooled to 0 ˚C. NaH (60% in mineral oil, 250 mg, 6.19 mmol, 3 equiv) was added to the mixture in 3 portions over 10 min. After 5 h the reaction was quenched by the careful addition of NH_4Cl_{aq} (sat), the layers were separated and the aqueous layer washed with ether (3x). The organic layers were combined, dried over $MgSO_4$, filtered and concentrated *in vacuo*. The crude oil was further purified by column chromatography (10% ether in pentane), which yielded the desired product as a thick clear oil (80%, 690 mg, 1.62 mmol).

^{1}H NMR (400 MHz, Chloroform-*d*) δ 7.39 – 7.22 (m, 10H), 6.91 – 6.78 (m, 3H), 4.71 (s, 2H), 4.54 (s, 2H), 4.48 (s, 2H), 3.88 (s, 3H), 3.83 (s, 3H), 3.85 – 3.79 (m, 1H), 3.74 – 3.47 (m, 4H).

^{13}C NMR (101 MHz, Chloroform-*d*) δ 149.1, 148.7, 138.8, 138.4, 131.0, 128.5, 128.4, 127.8, 127.7, 127.7, 127.6, 120.33, 111.1, 111.0, 77.4, 73.5, 73.4, 72.4, 70.5, 70.2, 56.1, 55.9.

$[\alpha]_D^{20}$= -1.0 (c = 0.1 in $CHCl_3$).

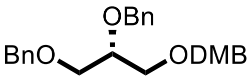

(*S*)-3-((3,4-dimethoxybenzyl)oxy)propane-1,2-di-benzylether (5'). This compound was prepared with the same synthetic procedure that was used for **5**

^{1}H NMR (400 MHz, Chloroform-*d*): Same as reported for compound **5**

^{13}C NMR (101 MHz, Chloroform-*d*): Same as reported for compound **5**

$[\alpha]_D^{20}$= +1.1 (c = 0.1 in $CHCl_3$).

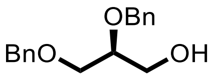

(S)-2,3-bis(benzyloxy)propan-1-ol (6). Dimethoxy-benzyl-di-benzyl glycerol (**5**) (220 mg, 0.52 mmol) was dissolved in CH_2Cl_2/H_2O (9:1, 6 mL) and cooled to 0 ˚C (ice/water bath). DDQ (142 mg, 0.625 mmol, 1.2 equiv) was added in 3 portions over 5 min. The resulting orange biphasic solution was allowed to stir for 3 h before filtration over celite. The filtrate was concentrated and further purified by column chromatography (20% EtOAc in pentane) to yield the desired alcohol as a clear thick oil (85%, 135 mg, 0.5 mmol).

^{1}H NMR (400 MHz, Chloroform-*d*) δ 7.45 – 7.28 (m, 10H), 4.73 (H_a, d, *J* = 11.7 Hz, 1H), 4.64 (H_b, d, *J* = 11.8 Hz, 1H), 4.61 – 4.52 (m, 2H), 3.86 – 3.53 (m, 5H), 2.30 (s, 1H).

^{13}C NMR (101 MHz, Chloroform-*d*) δ 138.4, 138.1, 128.5, 128.5, 127.9, 127.9, 127.8, 127.7, 78.2, 73.6, 72.2, 70.3, 62.9.

$[\alpha]_D^{20}$= -17.8 (c = 0.1 in $CHCl_3$).

HRMS-ESI+ (m/z): $[M + Na]^+$ calculated for $C_{17}H_{20}O_3Na$, 292.131; found, 292.130.

The spectral data correspond to previously reported (56)

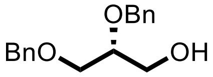

(S)-2,3-bis(benzyloxy)propan-1-ol (6'). This compound was prepared with the same synthetic procedure that was used for **6**

^{1}H NMR (400 MHz, Chloroform-*d*): Same as reported for compound **6**

^{13}C NMR (101 MHz, Chloroform-*d*): Same as reported for compound **6**

$[\alpha]_D^{20}$= +18.1 (c = 0.1 in $CHCl_3$).

HRMS-ESI+ (m/z): $[M + Na]^+$ calculated for $C_{17}H_{20}O_3Na$, 292.131; found, 292.130.

The spectral data correspond to previously reported (57)

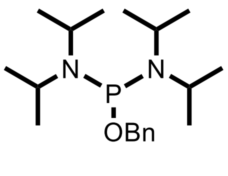

1-(benzyloxy)-N,N,N',N'-tetraisopropylphosphanediamine. To a stirring suspension of 1-chloro-N,N,N',N'-tetraisopropylphosphanediamine (1.33 g, 5 mmol) in dry Et_2O (20 mL) at 0 ˚C, was added a solution of benzyl alcohol (0.57 mL, 5.5 mmol, 1.1 equiv) and Et_3N (0.84 mL, 6 mmol, 1.2 equiv.) in dry Et_2O (5 mL) over 1/2 h (syringe pump). The resulting milky mixture was stirred for 3 h. The ammonium salts were filtered, the filtrate concentrated *in vacuo* and further purified by column chromatography (5% Et_3N in hexane) which yielded the desired phosphoramidite as a colourless oil (95%, 1.52 g, 4.49 mmol)

Spectral data were identical to previously reported (58)

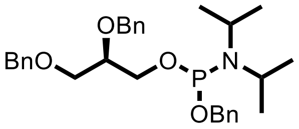

benzyl ((*R*)-2,3-bis(benzyloxy)propyl) diisopropylphosphoramidite (7). A flame dried Schlenk flask was charged with tetrazole (24 mg, 0.33 mmol, 0.95 eq), and to this was added dibenzylglycerol **6** (95 mg, 0.35 mmol) in dry toluene. The toluene was evaporated under Schlenk line vacuum while stirring vigorously, the co-evaporation with toluene was repeated three times. CH_2Cl_2 (1.2 mL) was added and the resulting solution was cooled to 0 ˚C, after which the benzyloxy-posphoramidite (237 mg, 0.7 mmol, 2 equiv) in CH_2Cl_2 (0.6 mL) was added in a dropwise fashion. After 16 h, all volatiles were evaporated *in vacuo* and the crude was further purified by column chromatography (10% ether, 5% Et_3N in pentane) which yielded the desired phosphoramidite as a thick oil. (95%, 170 mg, 0.33 mmol)

^{1}H NMR (400 MHz, Chloroform-*d*) δ 7.57 – 7.18 (m, 15H), 4.85 – 4.66 (m, 4H), 4.58 (t, *J* = 2.8 Hz, 2H), 3.92 – 3.81 (m, 2H), 3.82 – 3.74 (m, 1H), 3.75 – 3.55 (m, 4H), 1.29 – 1.15 (m, 12H).

^{13}C NMR (101 MHz, cdcl$_3$) δ 138.88, 138.51, 128.41, 128.36, 128.33, 127.84, 127.79, 127.70, 127.69, 127.59, 127.55, 127.32, 127.30, 127.06, 127.04, 78.17, 78.13, 78.09, 78.06, 73.46, 72.33, 70.56, 65.48, 65.30, 63.31, 63.24, 63.15, 63.08, 43.19, 43.06, 24.80, 24.76, 24.73, 24.68.

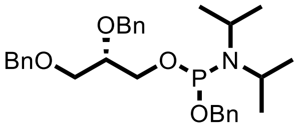

benzyl ((*S*)-2,3-bis(benzyloxy)propyl) diisopropylphosphoramidite (7'). This compound was prepared with the same synthetic procedure that was used for **7**

^{1}H NMR (400 MHz, Chloroform-*d*): Same as reported for compound **7**

^{13}C NMR (101 MHz, Chloroform-*d*): Same as reported for compound **7**

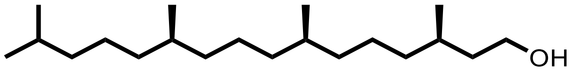

(3*R*,7*R*,11*R*)-3,7,11,15-tetramethylhexadecan-1-ol (9). To an oven dried 20 mL vial equipped with magnetic stirrer bar was added trans-phytol (1 g, 3.4 mmol) in 3 mL of dry methanol under nitrogen atmosphere. The resulting mixture was degassed by the freeze-pump-thaw technique after which (*S*)-BINAP-ruthenium(II) dicarboxylate (**II**) (6 mg, 0.07 mmol, 0.2 mol%) was added. The vial containing the reaction mixture was placed inside a 0.5 L Parr bomb and subjected to 75 bar of H_2 pressure for 72 h. The resulting brown solution was concentrated *in vacuo* and passed over a small silica column eluted with pentane. Evaporation of the solvent yielded diastereomerically pure (*R*,*R*,*R*)-phytanol as a yellow oil (96% yield, 0.97 g, d.r. >1:20, determined by NMR-spectroscopy).

^{1}H NMR (400 MHz, Chloroform-*d*) δ 3.76 – 3.61 (m, 2H), 1.99 (s, 1H), 1.73 – 1.45 (m, 3H), 1.45 – 0.96 (m, 21H), 0.95 – 0.76 (m, 15H).

^{13}C NMR (151 MHz, Chloroform-*d*) δ 61.2, 40.1, 39.5, 37.7, 37.6, 37.5, 37.5, 37.4, 32.9, 32.9, 29.7, 28.1, 24.9, 24.6, 24.5, 22.8, 22.7, 19.9, 19.9, 19.8.

$[\alpha]_D^{20}$= +1.90 (c = 0.1 in $CHCl_3$).

The spectral data correspond to previously reported.(50)

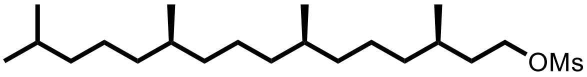

(3*R*,7*R*,11*R*)-3,7,11,15-tetramethylhexadecyl methanesulfonate (10). To a stirring solution of phytanol (2.99 g, 10 mmol) in CH_2Cl_2 (20 mL) at 0 ˚C (ice/water bath) was added Et_3N (4.2 mL, 30 mmol, 3 equiv), DMAP (611 mg, 5 mmol, 0.5 equiv) and MsCl (0.93 mL, 12 mmol, 1.2 equiv). After 2 h the mixture was quenched by the addition of $NaHCO_3$ (sat) and the aqueous layer was extracted with ether. All the organic layers were combined, dried over $MgSO_4$, filtered and concentrated *in vacuo*, the crude oil was further purified by column chromatography (15% ether in pentane) to give the desired mesylate as a pale oil (90%, 3.4 g, 8.95 mmol).

^{1}H NMR (400 MHz, Chloroform-*d*) δ 4.34 – 4.13 (m, 2H), 2.99 (s, 3H), 1.84 – 1.70 (m, 1H), 1.70 – 1.44 (m, 3H), 1.44 – 0.97 (m, 20H), 0.91 (d, *J* = 6.4 Hz, 3H), 0.88 – 0.80 (m, 12H).

^{13}C NMR (101 MHz, Chloroform-*d*) δ 68.7, 39.5, 37.5, 37.5, 37.5, 37.5, 37.4, 37.3, 37.2, 36.1, 32.9, 32.9, 29.5, 28.1, 24.9, 24.6, 24.3, 22.8, 22.7, 19.7, 19.4.

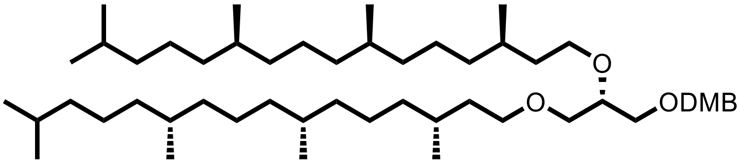

2,3-bisphytanol-*sn*-dimethoxybenzyl-glycerol (11). To a stirring solution of 15-crown-5 (0.55 mL, 2.8 mmol, 2.2 equiv) and sn-1-DMB-glycerol (200 mg, 0.83 mmol) in dry THF (1.2 mL) at 0 ˚C (ice/water bath) was added NaH (60% in mineral oil, 73mg, 1.81 mmol, 2.2 equiv) in 4 portions over 5 min. The mixture was allowed to warm up to rt over 1/2 h, after which mesylate **10** (685 mg, 1.82 mmol, 2.2 equiv) in dry THF (0.5 mL) was added in a dropwise fashion. After 16 h the reaction was quenched by the careful addition of NH_4Cl_{aq} (sat). The aqueous layer was extracted with Et_2O (3x), the organic layers combined, dried over $MgSO_4$, filtered and concentrated *in vacuo*. The crude oil was further purified by column chromatography (10% ether in pentane) which yielded the desired product as a pale yellow oil (40%, 267 mg, 3.33 mmol)

^{1}H NMR (400 MHz, Chloroform-*d*) δ 6.92 – 6.79 (m, 3H), 4.48 (s, 2H), 3.87 (s, 3H), 3.86 (s, 3H), 3.69 – 3.38 (m, 9H), 1.73 – 1.46 (m, 6H), 1.44 – 0.96 (m, 42H), 0.85 (m, 30H).

^{13}C NMR (101 MHz, Chloroform-*d*) δ 149.1, 148.6, 131.1, 120.3, 111.1, 111.0, 78.1, 73.4, 71.0, 70.2, 70.1, 69.0, 56.0, 55.9, 39.5, 37.7, 37.7, 37.6, 37.5, 37.5, 37.4, 37.3, 36.8, 33.0, 32.9, 30.1, 30.0, 28.1, 24.9, 24.6, 24.5, 22.9, 22.8, 19.9, 20.0, 19.9, 19.8.

HRMS-ESI+ (m/z): $[M + Na]^+$ calculated for $C_{52}H_{98}O_5Na$, 825.7307; found, 825.730.

$[\alpha]_D^{20}$= +0.70 (c = 0.1 in $CHCl_3$).

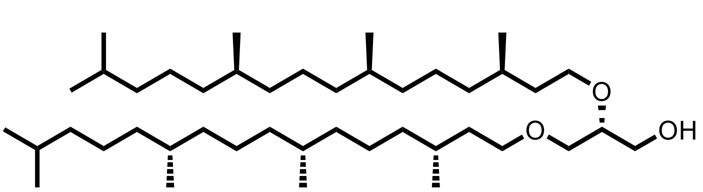

2,3-bisphytanol-*sn*-glycerol (11). 1-(3,4-dimethoxybenzyl)-2,3-bisphytanol-*sn*-glycerol (**12**) (400 mg, 0.6 mmol) was dissolved in CH_2Cl_2 (10 mL) to which water (1 mL) was added. The biphasic mixture was cooled to 0 °C (ice/water bath) and DDQ (136 mg, 0.6 mmol, 1.2 equiv) was added in three portions.

The resulting green biphasic mixture was stirred for 2 h at 0 °C during which it turned light brown. The mixture was quenched by the addition of $NaHCO_3$ (sat), the layers were separated and the aqueous layer was washed with ether (3x). The organic layers were combined, dried over $MgSO_4$, filtered and concentrated in vacuo. The crude oil was further purified by flash column chromatography (20% diethyl ether in pentane, I_2 and 2,4-DNP stain) which afforded the desired product as a viscous yellow oil (90%, 290 mg, 0.44 mmol).

^{1}H NMR (400 MHz, Chloroform-*d*) δ 3.78 – 3.40 (m, 9H), 2.21 (br s, 1H), 1.56 (m, 6H), 1.43 – 0.96 (m, 42H), 0.92 – 0.75 (m, 30H).

^{13}C NMR (101 MHz Chloroform-*d*) δ 78.5, 71.1, 70.3, 68.8, 63.2, 39.5, 37.6, 37.6, 37.5, 37.5, 37.5, 37.4, 37.2, 36.7, 32.9, 30.0, 30.0, 28.1, 24.9, 24.6, 24.5, 22.9, 22.8, 19.9, 19.9, 19.9, 19.8.

HRMS-ESI+ (m/z): $[M + H]^+$ calculated for $C_{43}H_{89}O_3H$, 653.6812; found, 653.679.

$[\alpha]_D^{20}$= +4.1 (c = 0.1 in $CHCl_3$).

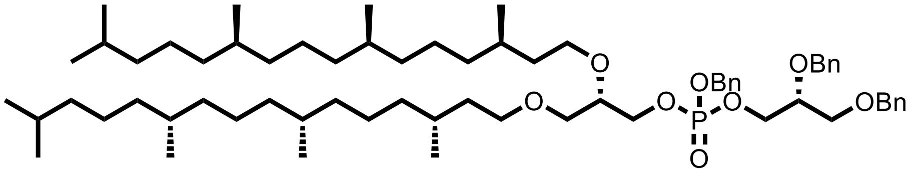

Benzyl (2,3-bisphytanol-*sn*-glycerol)-((*R*)-2,3-bis(benzyloxy)propyl) phosphate (13). 2,3-bisphytanol-*sn*-glycerol (20 mg, 30 µmol) and tetrazole (7 mg, 92 µmol, 3 equiv) were dissolved in dry toluene. The toluene was evaporated under Schlenk-line vacuum while stirring vigorously, this process was repeated three times. 200 µL of CH_2Cl_2 was added and the solution was cooled to 0 ˚C (ice/water bath). Phosphoramidite **7** (39 mg, 77 µmol, 2.5 equiv) in 100 µL CH_2Cl_2 was added in a drop-wise fashion and the mixture was stirred for 16 h during which it was allowed to warm up to rt. To the resulting mixture a 5M solution of *t*-BuOOH (60 µL,300 µmol, 10 eq) in decane was added in a drop-wise fashion and the mixture was allowed to stir for 30 minutes. The crude mixture was poured into phosphate buffer (1 M, pH = 7) and was extracted with Et_2O (3x). The organic extracts were combined, dried over $MgSO_4$, filtered and concentrated. The crude was further purified by column chromatography (20% ether in pentane) to give the pure protected glycerol-phosphate **13** as a yellow oil (75%, 25 mg, 23 µmol)

^{1}H NMR (400 MHz, Chloroform-*d*) δ 7.67 – 7.01 (m, 15H), 5.13 – 4.99 (m, 2H), 4.75 – 4.59 (m, 2H), 4.52 (d, 2H), 4.30 – 3.91 (m, 5H), 3.89 – 3.73 (m, 1H), 3.66 – 3.52 (m, 4H), 3.49 – 3.33 (m, 4H), 1.77 – 1.42 (m, 6H), 1.41 – 0.97 (m, 42H), 0.93 – 0.77 (m, 30H).

^{13}C NMR (101 MHz, Chloroform-*d*) δ 138.1, 137.98, 135.96, 135.9, 128.54, 128.53, 128.50, 128.47, 128.45, 128.43, 128.38, 128.3, 127.94, 127.85, 127.82, 127.80, 127.75, 127.7, 127.7, 127.6, 78.1, 77.3, 77.2, 76.6, 76.56, 76.52, 76.49, 73.4, 72.2, 70.1, 69.9, 69.8, 69.3, 69.23, 69.22, 69.21, 69.2, 69.0, 67.1, 67.1, 67.0, 66.9, 39.4, 37.6, 37.5, 37.5, 37.4, 37.3, 37.0, 36.6, 32.8, 30.3, 29.9, 29.8, 29.7, 28.0, 24.8, 24.5, 24.4, 22.7, 22.6, 19.8.

^{31}P NMR (162 MHz, Chloroform-*d*) δ -0.89,

HRMS-ESI+ (m/z): $[M + Na]^+$ calculated for $C_{67}H_{113}O_{8P}$, 1077.825; found, 1077.826.

$[\alpha]_D^{20}$= -3.7 (c = 0.1 in $CHCl_3$).

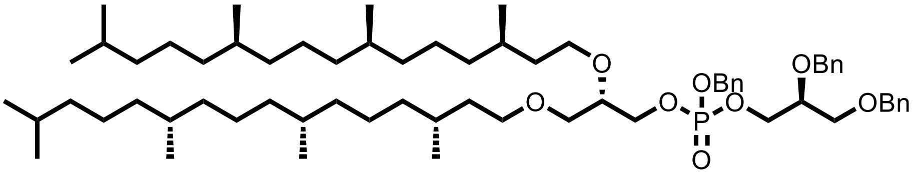

Benzyl (2,3-bisphytanol-*sn*-glycerol)-((*S*)-2,3-bis(benzyloxy)propyl) phosphate (13').This compound was prepared with the same synthetic procedure that was used for **13**

^{1}H NMR (400 MHz, Chloroform-*d*) δ 7.67 – 7.01 (m, 15H), 5.13 – 4.99 (m, 2H), 4.75 – 4.59 (m, 2H), 4.52 (d, 2H), 4.30 – 3.91 (m, 5H), 3.89 – 3.73 (m, 1H), 3.66 – 3.52 (m, 4H), 3.49 – 3.33 (m, 4H), 1.77 – 1.42 (m, 6H), 1.41 – 0.97 (m, 42H), 0.93 – 0.77 (m, 30H).

^{13}C NMR (101 MHz, Chloroform-*d*) δ 138.1, 137.98, 135.96, 135.9, 128.54, 128.53, 128.50, 128.47, 128.45, 128.43, 128.38, 128.3, 127.94, 127.85, 127.82, 127.80, 127.75, 127.7, 127.7, 127.6, 78.1, 77.3, 77.2, 76.6, 76.56, 76.52, 76.49, 73.4, 72.2, 70.1, 69.9, 69.8, 69.3, 69.23, 69.22, 69.21, 69.2, 69.0, 67.1, 67.1, 67.0, 66.9, 39.4, 37.6, 37.5, 37.5, 37.4, 37.3, 37.0, 36.6, 32.8, 30.3, 29.9, 29.8, 29.7, 28.0, 24.8, 24.5, 24.4, 22.7, 22.6, 19.8.

^{31}P NMR (162 MHz, Chloroform-*d*) δ -0.89,

HRMS-ESI+ (m/z): $[M + Na]^+$ calculated for $C_{67}H_{113}O_{8P}$, 1077.825; found, 1077.827.

$[\alpha]_D^{20}$= +3.60 (c = 0.1 in $CHCl_3$).

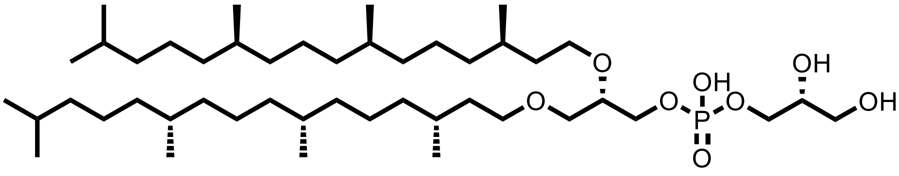

2,3-bisphytanol-*sn*-glycerol-((R)-2,3-dihydroxypropyl) hydrogen phosphate (14) (Figure S5). 13 was dissolved in a mixture of ethanol/THF (1:1) and was degassed via the freeze-pump-thaw technique. $Pd(OH)_2$/C (20%, 8.5 mg, 12 µmol, 1 eq) was added to the stirring solution under N_2-atmosphere. The resulting suspension was placed under H_2 atmosphere and was allowed to stir for 16 h at rt. The mixture was filtered over celite and further purified by column chromatography (100% $CHCl_3$ → 30% MeOH in $CHCl_3$, Davisil Grade 635 silica) which yielded the desired pospholipid **14** as a white film (71%, 7 mg, 9 µmol).

^{1}H NMR (400 MHz, Methanol-*d*$_4$) δ 4.05 – 3.82 (m, 2H), 3.75 (p, *J* = 6.8 Hz, 4H), 3.67 – 3.48 (m, 5H), 3.49 – 3.38 (m, 2H), 3.38 – 3.27 (m, 1H), 2.68 (s, 1H), 2.39 (s, 1H), 1.60 – 1.42 (m, 5H), 1.41 – 1.12 (m, 24H), 1.13 – 0.95 (m, 32H), 0.89 – 0.75 (m, 18H).

^{13}C NMR (151 MHz, MeOD) δ 71.5, 71.0, 70.7, 69.5, 67.0, 65.6, 62.8, 58.1, 39.9, 37.9, 37.8, 37.5, 37.2, 33.3, 32.4, 30.5, 30.4, 30.2, 29.8, 28.5, 26.0, 25.3, 25.0, 24.9, 23.1, 23.0, 20.15, 20.06.

^{31}P NMR (243 MHz, MeOD) δ -0.41.

HRMS-ESI- (m/z): $[M - H]^-$ calculated for $C_{46}H_{94}O_8P$, 805.669; found, 805.669.

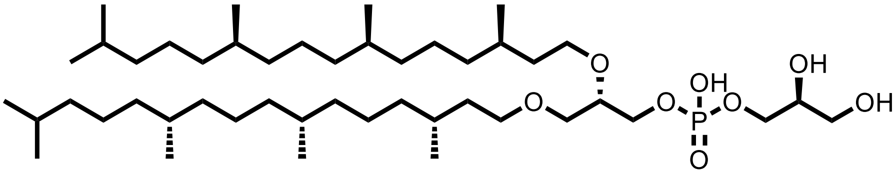

2,3-bisphytanol-*sn*-glycerol-((S)-2,3-dihydroxypropyl) hydrogen phosphate (14') (Figure S6). This compound was prepared with the same synthetic procedure that was used for **14**.

^{1}H NMR (400 MHz, Methanol-d_4) δ 4.05 – 3.82 (m, 2H), 3.75 (p, *J* = 6.8 Hz, 4H), 3.67 – 3.48 (m, 5H), 3.49 – 3.38 (m, 2H), 3.38 – 3.27 (m, 1H), 2.68 (s, 1H), 2.39 (s, 1H), 1.60 – 1.42 (m, 5H), 1.41 – 1.12 (m, 24H), 1.13 – 0.95 (m, 32H), 0.89 – 0.75 (m, 18H).

^{13}C NMR (151 MHz, MeOD) δ 71.5, 71.0, 70.7, 69.5, 67.0, 65.6, 62.8, 58.1, 39.9, 37.9, 37.8, 37.5, 37.2, 33.3, 32.4, 30.5, 30.4, 30.2, 29.8, 28.5, 26.0, 25.3, 25.0, 24.9, 23.1, 23.0, 20.15, 20.06.

^{31}P NMR (243 MHz, MeOD) δ -0.41.

HRMS-ESI- (m/z): $[M - H]^-$ calculated for $C_{46}H_{94}O_8P$, 805.669; found, 805.669.

A

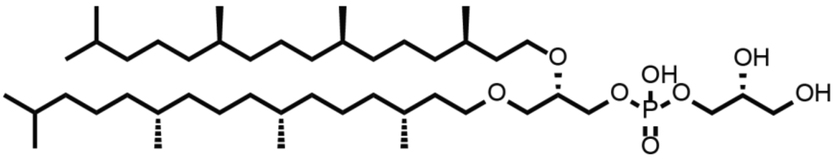
OH
OH
O
O
O
P
O
O
OH

B

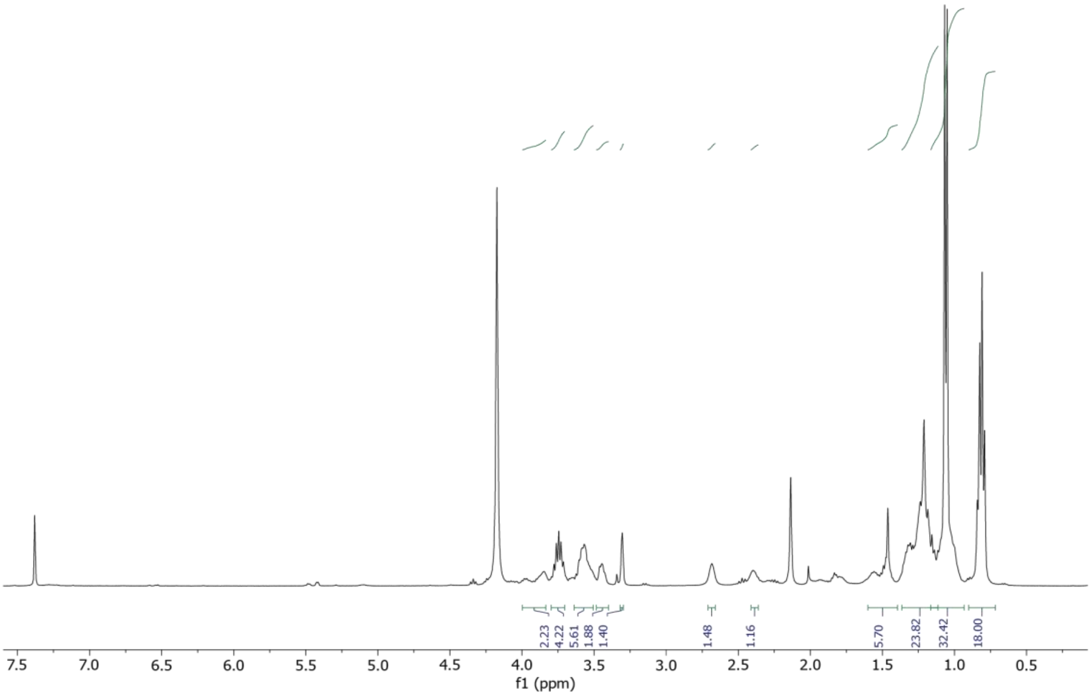
2.23
4.22
5.61
1.88
1.40
1.48
1.16
5.70
23.82
32.42
18.00
7.5 7.0 6.5 6.0 5.5 5.0 4.5 4.0 3.5 3.0 2.5 2.0 1.5 1.0 0.5
f1 (ppm)

C

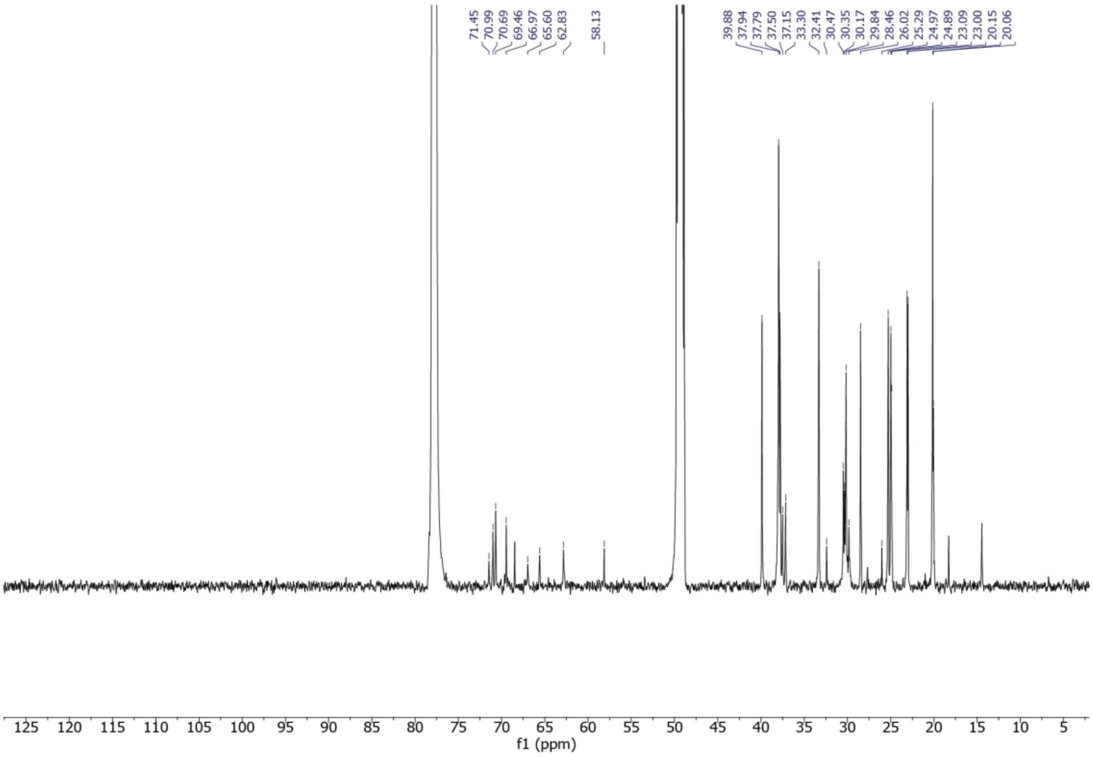
71.45
70.99
70.69
69.46
66.97
65.60
62.83
58.13
39.88
37.94
37.79
37.50
37.15
33.30
32.41
30.47
30.35
30.17
29.84
28.46
26.02
25.29
24.97
24.89
23.09
23.00
20.15
20.06
125 120 115 110 105 100 95 90 85 80 75 70 65 60 55 50 45 40 35 30 25 20 15 10 5
f1 (ppm)

D

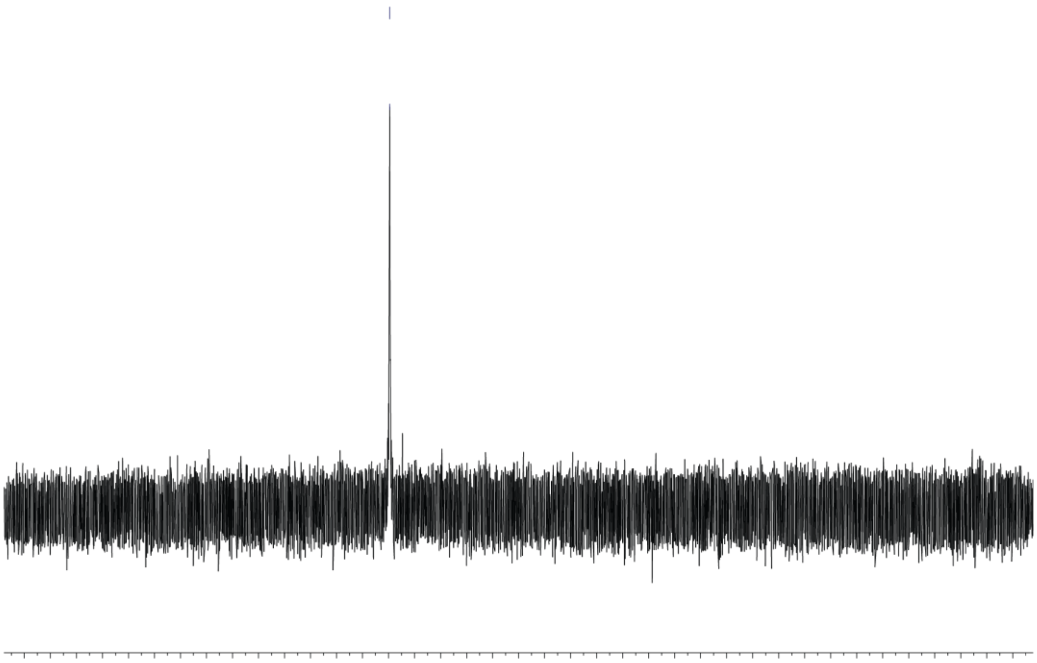

E

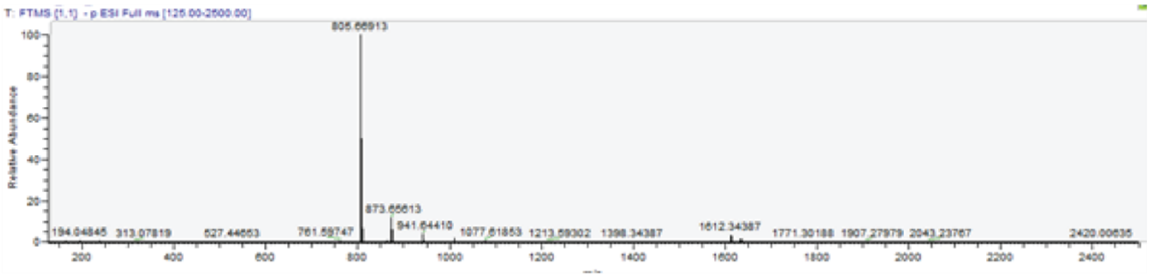

Figure S5: 2,3-bisphytanol-*sn*-glycerol-((R)-2,3-dihydroxypropyl) hydrogen phosphate (14) (A) Structure of **14**. **(B)** ^{1}H-NMR of **14**. **(C)** ^{13}C-NMR of **14**. **(D)** ^{31}P-NMR of **14**. **(E)** HRMS of **14**.

A

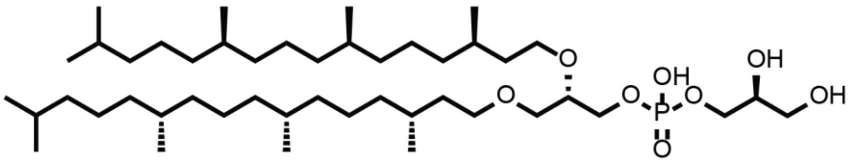

B

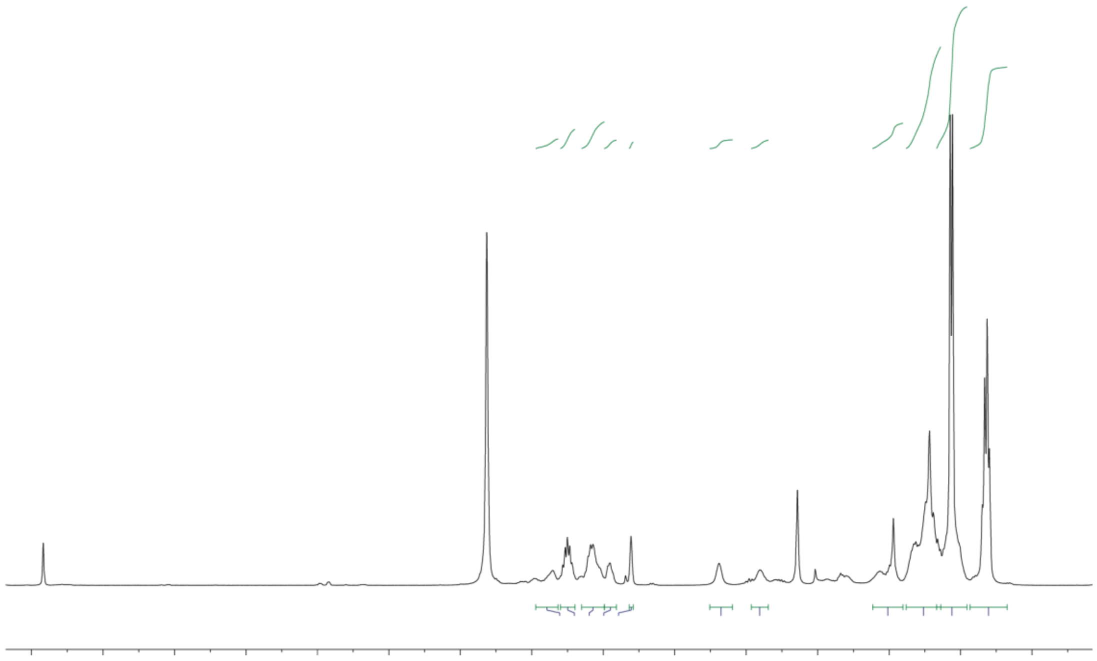

C

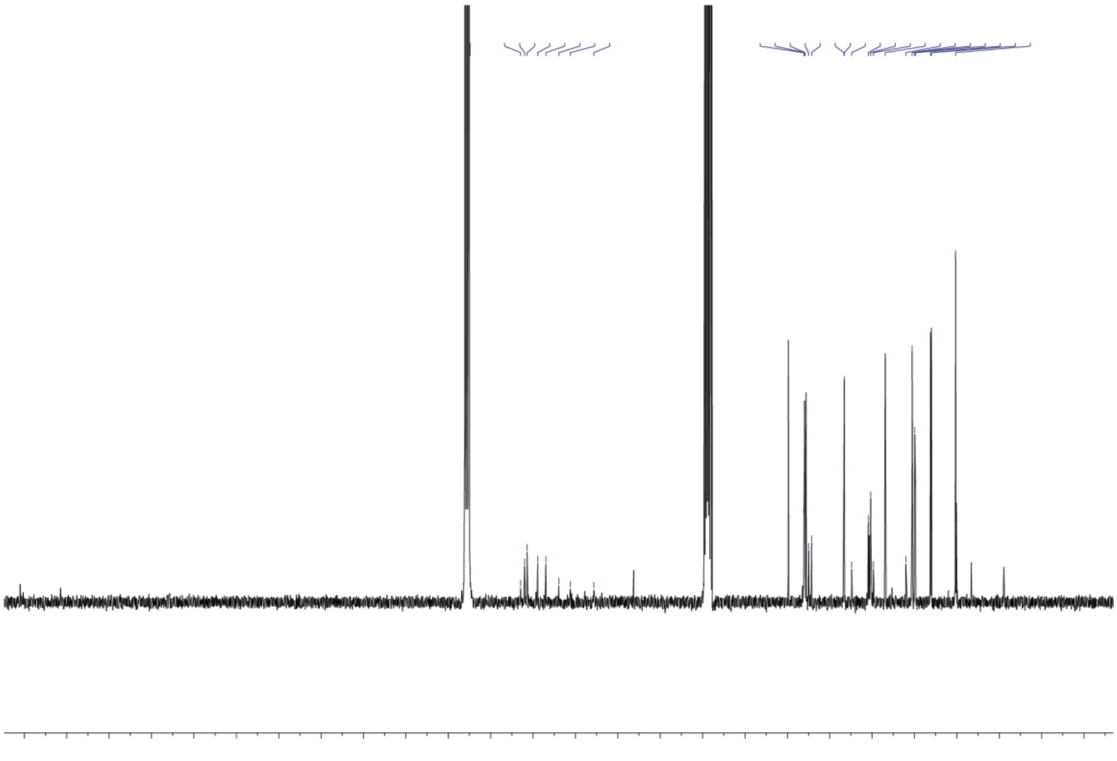

D

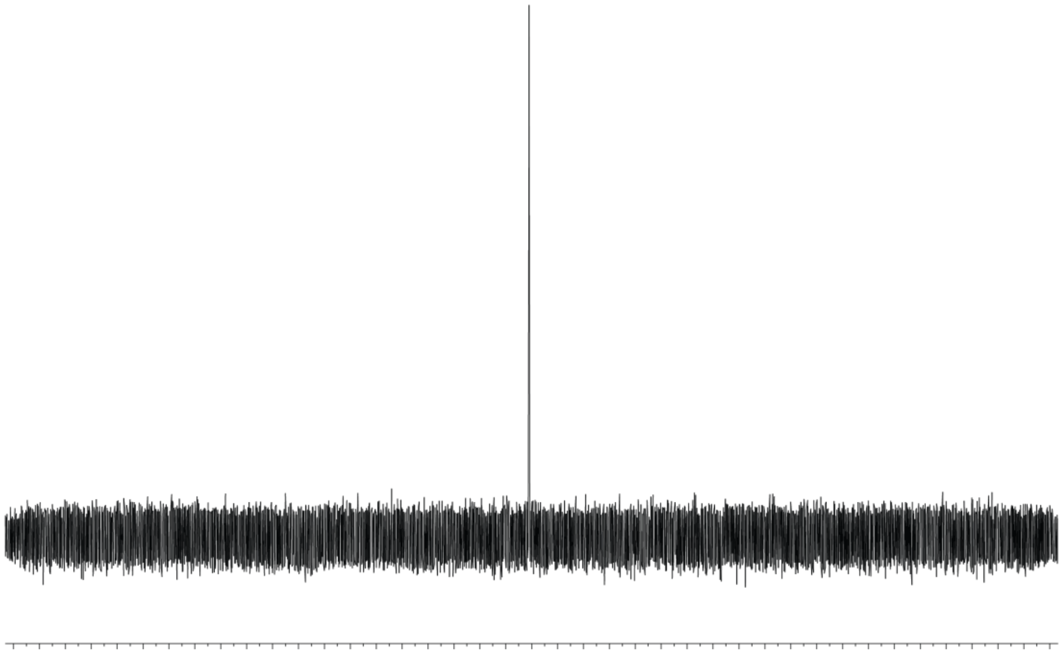

E

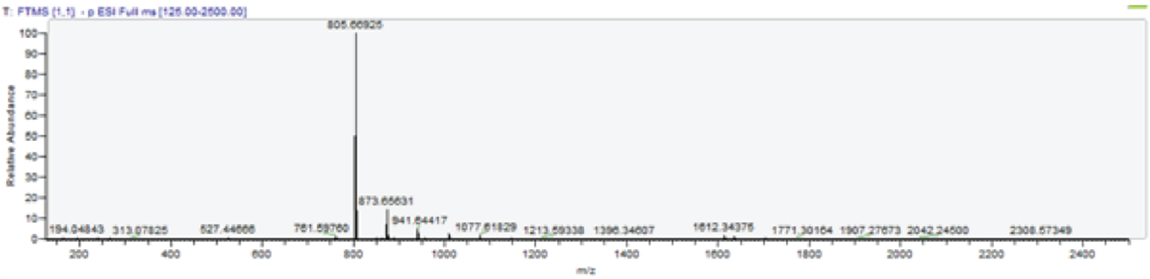

Figure S6: 2,3-bisphytanol-*sn*-glycerol-((S)-2,3-dihydroxypropyl) hydrogen phosphate (14’) (A) Structure of **14’**. **(B)** ^{1}H-NMR of **14’**. **(C)** ^{13}C-NMR of **14’**. **(D)** ^{31}P-NMR of **14’**. **(E)** HRMS of **14’**.

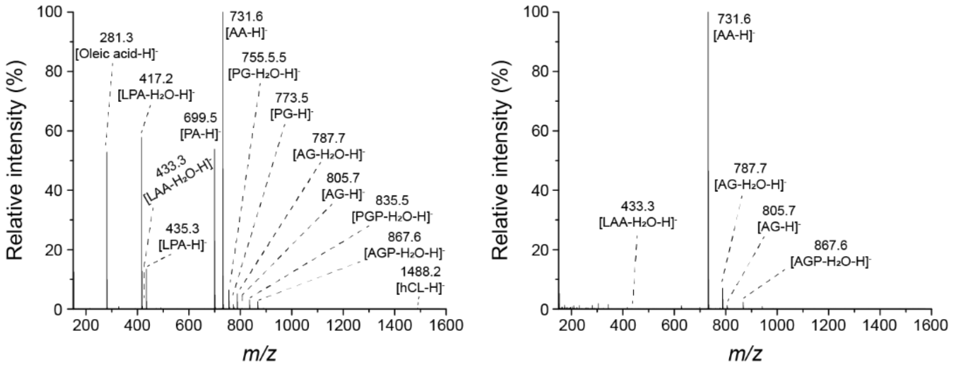

Figure S7. LC-MS fragmentation spectra of hybrid archaetidyl-phosphatidyl-cardiolipin (APCL; left panel) and di-archaetidyl-cardiolipin (DACL; right panel). Terms: lyso-phosphatidic acid (LPA), lyso-archaetidic acid (LAA), phosphatidic acid (PA), archaetidic acid (AA), phosphatidylglycerol (PG), archaetidylglycerol (AG), phosphatidylglycerol-phosphate (PGP), archaetidylglycerol-phosphate (AGP), hybrid cardiolipin (hCL; also referred to as APCL).

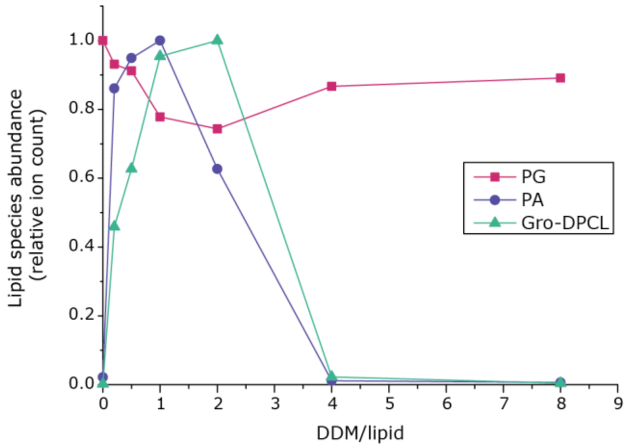

Figure S8. n-Dodecyl β-D-maltoside (DDM)-dependence of the activity of MhCls. The activity of MhCls was tested with different DDM-lipid molar ratios. Individual lipid species phosphatidylglycerol (PG), phosphatidic acid (PA) and glycerol-di-phosphatidyl-cardiolipin (Gro-DPCL) were analyzed by LC-MS, and plotted on the Y-axis relative to the highest detected amount of that particular lipid species.

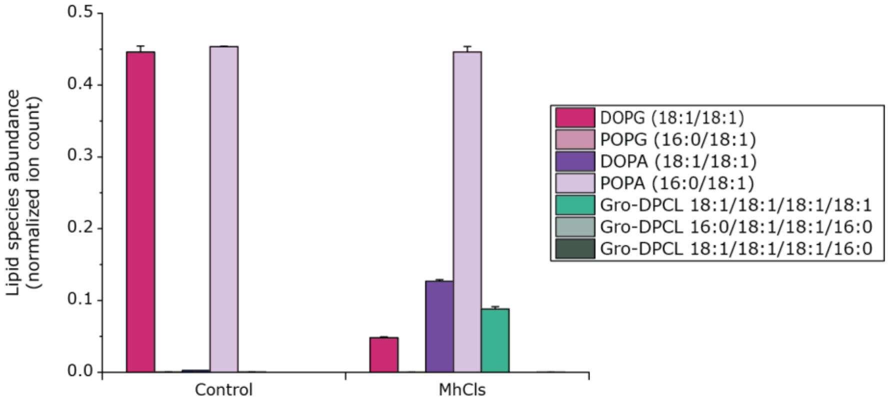

Figure S9. MhCls activity in the presence of DOPG and POPA. Lipid species di-oleoyl-phosphatidylglycerol (DOPG), palmitoyl-oleoyl-phosphatidylglycerol (POPG), di-oleoyl-phosphatidic acid (DOPA), palmitoyl-oleoyl-phosphatidic acid (POPA) and the glycerol-diphosphatidyl-cardiolipin species (Gro-DPCL), were analyzed by LC-MS, normalized for the internal standard DDM and plotted.

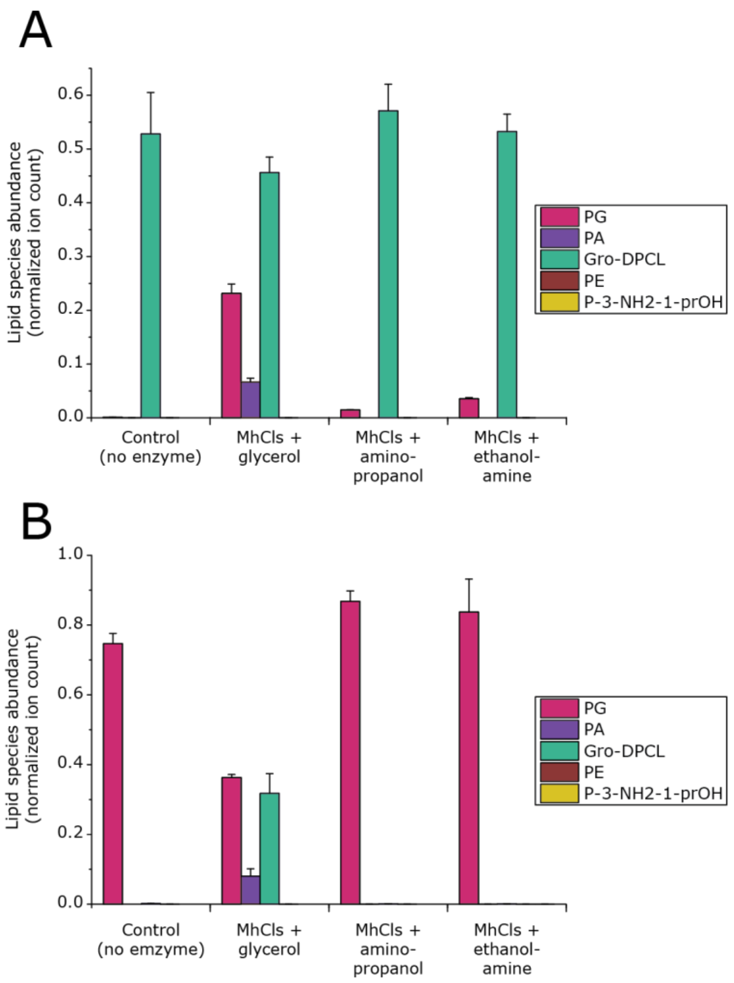

Figure S10. **MhCls activity in the presence of glycerol and the amines 3-amino-propanol and ethanolamine.** Lipid species phosphatidylglycerol (PG), phosphatidic acid (PA), glycerol-di-phosphatidyl-cardiolipin (Gro-DPCL), phosphatidylethanolamine (PE) and phosphatidyl-3-amino-propanol (P-3-NH2-1-PrOH) were analyzed by LC-MS, normalized for the internal standard DDM, and plotted.

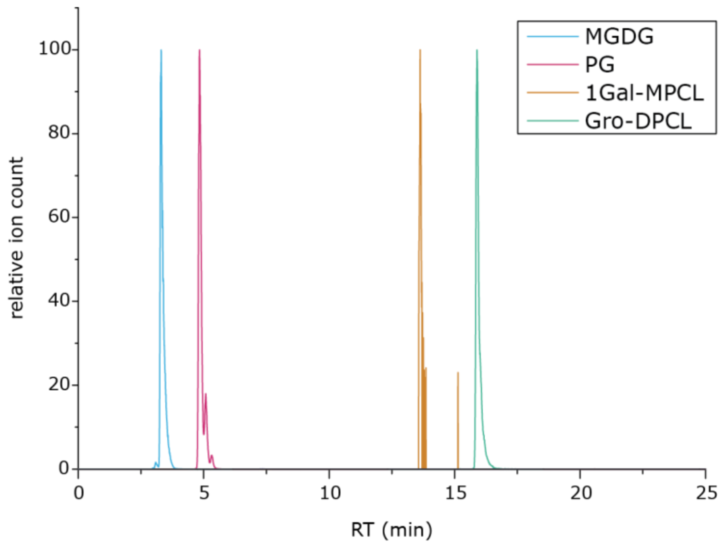

Figure S11. **Retention times of the lipid species mono-galactosyl-diacylglycerol (MGDG), phosphatidylglycerol (PG), 1-galactosyl-mono-phosphatidyl-cardiolipin (1Gal-MPCL) and glycerol-di-phosphatidyl-cardiolipin (Gro-DPCL), during LC-MS analysis.** The extracted ion chromatogram peaks are all normalized to 100%.

Materials and methods

Bioinformatic identification of bacterial homologs of *E. coli* ClsA.
Using *E. coli* K12; MG1655 ClsA (Ec_ClsA: NP_415765.1) as the query sequence, BLAST homology searches to the domain of bacteria were performed, and sequences were selected from the classes Gammaproteobacteria, Deltaproteobacteria and Betaproteobacteria (all belonging to the phylum proteobacteria), to get a versatile group of bacterial ClsA. The BLAST results were further analyzed using MEGA X, where the sequences were combined with the group of archaeal methanogens (Figure 2C) and aligned using the MUSCLE algorithm (default settings).

Assay conditions. The detergent-dependent activity of MhCls was assayed in 300 mM MES pH 7.0, 100mM KCl, in the presence of 1 μM MhCls and 1 mM DOPG, for 6 minutes at 37°C. The glycerol-dependent dynamic equilibrium of MhCls in the presence of POPA was assayed in presence of, 1.8 mM DDM, and 0.5 mM DOPG together with 0.5 mM POPA. The activity of MhCls in the presence of glycerol and the amines 3-amino-1-propanol and ethanolamine was tested using either 100 mM Glycerol, 3-amino-1-propanol, or ethanolamine and 0.5 mM Gro-DPCL or 1 mM DOPG, with addition of 1.8 mM DDM.

References

47. Hall, B. G. (2013) Building phylogenetic trees from molecular data with MEGA. *Mol. Biol. Evol.* **30**, 1229–1235
50. Sita, L. R. (1993) Convenient highly stereoselective syntheses of (3R,7R,11R)- and (3S,7R,11R)-3,7,11,15-tetramethylhexadecanoic acid (phytanic acid) and the corresponding 3,7,11,15-tetramethylhexadecan-1-ols. *J. Org. Chem.* **58**, 5285–5287
51. Aspinall, H. C., Greeves, N., Lee, W.-M., McIver, E. G., and Smith, P. M. (1997) An improved Williamson etherification of hindered alcohols promoted by 15-crown-5 and sodium hydride. *Tetrahedron Lett.* **38**, 4679–4682
52. Yeh, E. A.-H., Kumli, E., Damodaran, K., and Curran, D. P. (2013) Bare-minimum fluorous mixture synthesis of a stereoisomer library of 4,8,12-trimethylnonadecanols and predictions of NMR spectra of saturated oligoisoprenoid stereoisomers. *J. Am. Chem. Soc.* **135**, 1577–1584
53. Smith, A. B., and Safonov, I. G. (2002) Total Synthesis of (+)-Dactylolide. *Org. Lett.* **4**, 635–637
54. Schaus, S. E., Brandes, B. D., Larrow, J. F., Tokunaga, M., Hansen, K. B., Gould, A. E., Furrow, M. E., and Jacobsen, E. N. (2002) Highly Selective Hydrolytic Kinetic Resolution of Terminal Epoxides Catalyzed by Chiral (salen)CoIII Complexes. Practical Synthesis of Enantioenriched Terminal Epoxides and 1,2-Diols. *J. Am. Chem. Soc.* **124**, 1307–1315
55. Alcaraz, M.-L., Peng, L., Klotz, P., and Goeldner, M. (1996) Synthesis and Properties of Photoactivatable Phospholipid Derivatives Designed To Probe the Membrane-Associate Domains of Proteins. *J. Org. Chem.* **61**, 192–201
56. Keith, D. J., and Townsend, S. D. (2019) Total Synthesis of the Congested, Bisphosphorylated *Morganella morganii* Zwitterionic Trisaccharide Repeating Unit. *J. Am. Chem. Soc.* **141**, 12939–12945
57. Sung'hwa, F., Strik, A., Regeling, H., Zwanenburg, B., and Chittenden, G. J. F. (2006) Synthesis of 2(R),3-dihydroxypropyl and 2(R),3(R)-dihydroxybutyl beta-D-fructopyranosides and some derivatives. *Carbohydr. Res.* **341**, 846–854
58. Iwashita, M., Makide, K., Nonomura, T., Misumi, Y., Otani, Y., Ishida, M., Taguchi, R., Tsujimoto, M., Aoki, J., Arai, H., and Ohwada, T. (2009) Synthesis and Evaluation of Lysophosphatidylserine Analogues as Inducers of Mast Cell Degranulation. Potent Activities of Lysophosphatidylthreonine and Its 2-Deoxy Derivative. *J. Med. Chem.* **52**, 5837–5863
